# Skeletal Muscle Mitochondrial and Autophagic Dysregulation Are Modifiable in Spinal Muscular Atrophy

**DOI:** 10.1002/jcsm.13701

**Published:** 2025-02-03

**Authors:** Andrew I. Mikhail, Sean Y. Ng, Donald Xhuti, Magda A. Lesinski, Jennifer Chhor, Marc‐Olivier Deguise, Yves De Repentigny, Joshua P. Nederveen, Rashmi Kothary, Mark A. Tarnopolsky, Vladimir Ljubicic

**Affiliations:** ^1^ Department of Kinesiology McMaster University Hamilton Ontario Canada; ^2^ Department of Pediatrics McMaster University Medical Center Hamilton Ontario Canada; ^3^ Regenerative Medicine Program Ottawa Hospital Research Institute Ottawa Ontario Canada; ^4^ Faculty of Medicine University of Ottawa Ottawa Ontario Canada; ^5^ Division of Neonatology, Department of Pediatrics Children's Hospital of Eastern Ontario Ottawa Ontario Canada; ^6^ Department of Medicine University of Ottawa Ottawa Ontario Canada; ^7^ Department of Cellular and Molecular Medicine University of Ottawa Ottawa Ontario Canada; ^8^ Canada and Centre for Neuromuscular Disease University of Ottawa Ottawa Ontario Canada

**Keywords:** autophagy, biogenesis, dynamics, exercise, mitophagy, urolithin A

## Abstract

**Background:**

Spinal muscular atrophy (SMA) is a health‐ and life‐limiting neuromuscular disorder. Although varying degrees of mitochondrial abnormalities have been documented in SMA skeletal muscle, the influence of disease progression on pathways that govern organelle turnover and dynamics are poorly understood. Thus, the purpose of this study was to investigate skeletal muscle mitochondria during SMA disease progression and determine the effects of therapeutic modalities on organelle biology.

**Methods:**

*Smn* ^*2B/+*^ and *Smn* ^*2B/−*^ severe SMA‐like mice were used to investigate mitochondrial turnover and dynamics signalling. Muscles were analysed at postnatal day 9 (P9), P13 or P21 to address pre‐symptomatic, early symptomatic and late symptomatic periods of the disorder. Additionally, we utilized an acute dose of exercise and urolithin A (UA) to stimulate organelle remodelling in skeletal muscle of SMA mice in vivo and in SMA patient‐derived myotubes in vitro, respectively.

**Results:**

*Smn* ^*2B/+*^ and *Smn* ^*2B/−*^ mice demonstrated similar levels of muscle mitochondrial oxidative phosphorylation (OxPhos) proteins throughout disease progression. In contrast, at P21 the mRNA levels of upstream factors important for the transcription of mitochondrial genes encoded by the nuclear and mitochondrial DNA, including *nuclear respiratory factor 2*, *sirtuin 1*, *mitochondrial transcription factor A* and *tumour protein 53*, were upregulated (+31%–195%, *p* < 0.05) in *Smn* ^*2B/−*^ mice relative to *Smn* ^*2B/+*^. Early and late symptomatic skeletal muscle from SMA‐like mice showed greater autophagosome formation as denoted by more phosphorylated autophagy related 16‐like 1 (p‐ATG16L1^Ser278^) puncta (+60%–80%, *p* < 0.05), along with a build‐up of molecules indicative of damaged mitochondria such as BCL2 interacting protein 3, Parkin and PTEN‐induced kinase 1 (+100%–195%, *p* < 0.05). Furthermore, we observed a fragmented mitochondrial phenotype at P21 that was concomitant with abnormal splicing of *Optic atrophy 1* transcripts (−53%, *p* < 0.05). A single dose of exercise augmented the expression of *citrate synthase* (+43%, *p* < 0.05) and corrected the over‐assembly of autophagosomes (−64%, *p* < 0.05). In patient muscle cells, UA treatment stimulated autophagic flux, increased the expression of OxPhos proteins (+15%–47%, *p* < 0.05) and improved maximal oxygen consumption (+84%, *p* < 0.05).

**Conclusions:**

Abnormal skeletal muscle mitochondrial turnover and dynamics are associated with disease progression in *Smn* ^*2B/−*^ mice despite compensatory elevations in upstream factors important for organelle synthesis and recycling. Exercise and UA enhance mitochondrial health in skeletal muscle, which indicates that lifestyle‐based and pharmacological interventions may be effective countermeasures targeting the organelle for therapeutic remodelling in SMA.

## 1 Introduction

Spinal muscular atrophy (SMA) is a monogenic, life‐limiting neuromuscular disorder (NMD), a leading genetic cause of infant mortality, and among the most prevalent autosomal recessive conditions [1]. A homozygous mutation or deletion of the telomeric *Survival Motor Neuron 1* (*SMN1*) gene results in the depletion of functional SMN protein, which is a key molecule involved in numerous critical roles [1]. Although deterioration of spinal cord ⍺‐motoneurons has historically been considered the primary cause of the progressive denervation of myofibres and skeletal muscle atrophy, a growing body of literature affirms the skeletal muscle‐specific involvement of SMA independent of the anterograde effects of ⍺‐motoneurons [2, 3, 4, 5]. In many jurisdictions, there are three approved therapies for SMA that all work by specifically boosting SMN protein expression, which improves health and extends survival in affected individuals [1]. Unfortunately, these countermeasures are not cures and fail to address secondary consequences of the disorder, including skeletal muscle impairments [6]. Thus, it is important to examine the cell autonomous mechanisms that lead to functional deficits of skeletal muscle in SMA to better understand the pathology and to develop new, effective therapies.

Mitochondria are plastic organelles that undergo constant morphological and functional remodelling through turnover (i.e., biogenesis and mitophagy) and dynamics (i.e., fusion and fission) [7]. A healthy pool of these organelles is important for maintaining overall health of the neuromuscular system by regulating skeletal muscle mass and function [8], maintaining neuromuscular junction stability and protecting against ⍺‐motoneuron degeneration [9, 10]. Some evidence suggests that individuals with SMA display severe blunting of skeletal muscle mitochondrial content and respiratory activity [11, 12], whereas other results have demonstrated similar expression of organelle markers between SMA patients and healthy individuals [13, 14]. These inconsistencies could be partially attributed to differences in mitochondrial markers investigated (e.g., content vs. enzyme activity), as well as disease severity because the lowest levels of mtDNA were observed in the most severe Type I SMA [11]. Furthermore, there is currently a limited understanding of the consequence of SMA disease progression on key pathways that dictate skeletal muscle mitochondrial health. It is also unclear if addressing the dysregulation in mitochondrial turnover and dynamics could remedy skeletal muscle health in SMA, as it does in other conditions of neuromuscular deterioration [9, 15]. In the present study, we aimed to comprehensively investigate the molecular and morphological consequences of SMA disease progression on skeletal muscle mitochondria. Furthermore, we assess the efficacy of translational, physiological and pharmacological approaches to correct abnormal mitochondrial‐ and autophagy‐related signalling.

## 2 Methods

### 2.1 Ethical Approval

All experiments conducted in this study were approved by the Animal Research Ethics Board at McMaster University in accordance with guidelines set forth by the Canadian Council on Animal Care, are listed in the investigators' Animal Utilization Protocol no. 22‐07‐26 and have therefore been performed in accordance with the ethical standards laid down in the 1964 Declaration of Helsinki and its later amendments.

### 2.2 Animals

*Smn* ^*2B/2B*^ animals were crossed with heterozygous *Smn* ^*+/−*^ mice to generate healthy *Smn* ^*2B/+*^ and *Smn* ^*2B/−*^ (SMA‐like) mice, which more closely resemble a Type II SMA phenotype as previously reported [16, 17]. Male and female mice were utilized during this study due to similarities in phenotype and disease severity over time [S1]. To assess mitochondrial biology through the course of the disease progression, *Smn* ^*2B/+*^ and *Smn* ^*2B/−*^ littermates were sacrificed via cervical dislocation at postnatal day 9 (P9), P13 or P21 to resemble pre‐symptomatic, early symptomatic and late symptomatic stages. For experiments utilizing a single dose of exercise, tissues were collected from P17 sedentary WT (*Smn* ^*2B/+*^‐SED) and *Smn* ^*2B/−*^‐SED mice as well as from *Smn* ^*2B/−*^ animals immediately after exercise (*Smn* ^*2B/−*^‐0h), or 3‐h post‐exercise (*Smn* ^*2B/−*^‐3h). The triceps brachii (TRI), tibialis anterior (TA) and extensor digitorum longus (EDL) were collected from the same animal and processed for protein, mRNA and histological assays, respectively. To reduce variability between measures, the TRI, TA and EDL were chosen for analysis due to their similar contractile and metabolic characteristics as predominantly fast twitch muscles [S2], as well as due to their extensive involvement and recruitment during treadmill running exercise [S3–S5]. It is important to note that these muscles may each demonstrate unique responses to the same stimulus. During all experiments, animals were housed under a 12‐h light/dark cycle and provided food and water *ad libitum*.

### 2.3 Exercise Protocol and Tissue Collection

P17 *Smn* ^*2B/+*^ and *Smn* ^*2B/−*^ mice were randomly assigned to either an SED group or exercised to exhaustion on a motorized treadmill, and tissues were harvested immediately after (*Smn* ^*2B/−*^‐0h) or following a 3‐h recovery period (*Smn* ^*2B/−*^‐3h). Additional information can be found in the Supporting Information.

### 2.4 Cell Culture, Urolithin A (UA) Treatment and Metabolic Measurements of Human Immortalized SMA Myotubes

Immortalized human skeletal muscles cells from a paravertebral muscle biopsy of an 11‐year‐old SMA patient (0 *SMN1* and 3 *SMN2* copies; KM432‐7PV) were a kind gift from Dr Vincent Mouly (Institute of Myology, Paris, France). The paravertebral muscles are predominantly a type 1 phenotype and may exhibit different properties compared to other fast twitch muscles used in animal experiments. All cell culture experiments were performed as outlined in the Supporting Information.

### 2.5 Quantification of Protein and mRNA Expression

Details regarding protein extraction and western blotting on the TRI muscles as well as quantitative real‐time polymerase chain reaction (qPCR) on the TA are listed in the Supporting Information. A list of the primary antibodies and primers used can be found in Table S1 and Table S2, respectively.

### 2.6 Histochemical Staining, Immunofluorescence Microscopy and Analysis

All staining procedures were performed on 10‐μm‐thick EDL cross‐sections as described in the Supporting Information. A list of the antibodies utilized can be found in Table S1.

### 2.7 Transmission Electron Microscopy (TEM) and Analysis

TA samples from *Smn* ^*2B/+*^ and *Smn* ^*2B/−*^ mice were processed for transmission electron microscopy (TEM) as previously described [17]. Mitochondrial morphological assessments in the present study were done using TEM images of intermyofibrillar mitochondria that were previously captured by Deguise et al. [17]. Quantitative measurements were performed using ImageJ software and details regarding the computation of the aspect ratio, form factor, circularity, and roundness can be found in the Supporting Information.

### 2.8 Statistical Analysis

A two‐way analysis of variance (ANOVA) was employed to assess main effects of age and genotype. When appropriate, a Tukey's post hoc test was performed to determine groups that are significantly different. A two‐tailed independent Student's *t*‐test was employed for TEM metrics to determine differences between genotypes. A log‐rank test was utilized to determine significance in total distance travelled during exercise between genotypes, and a one‐way ANOVA was utilized to determine significance following a single dose of exercise. GraphPad Prism software version 9.1.1 was used for statistical analyses. All data are expressed as mean ± SEM. Statistical significance was accepted at *p* < 0.05.

## 3 Results

### 3.1 SMA‐Associated Myofibre Atrophy and Denervation Occur During Late Symptomatic Stages

**FIGURE 1:**
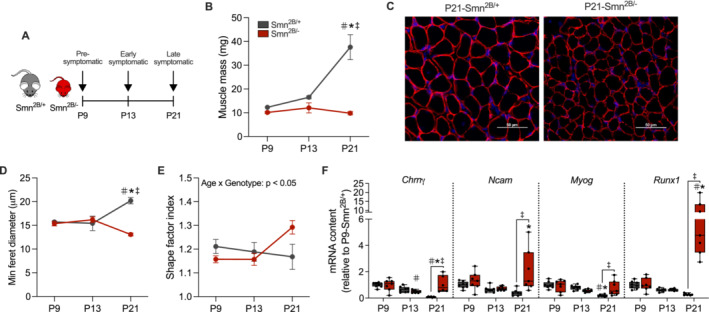
Time‐dependent changes in myofibre morphology and denervation in a mouse model of SMA (A) Schematic of the experimental design. Skeletal muscle samples were collected from *Smn* ^*2B/+*^ and *Smn* ^*2B/−*^ mice at postnatal day 9 (P9), P13 and P21 to represent pre‐symptomatic, early symptomatic and late symptomatic stages, respectively. (B) Summary of triceps brachii (TRI) muscle mass of *Smn* ^*2B/+*^ and *Smn* ^*2B/−*^ mice. *n* = 3–8. (C) Immunofluorescence microscopy of 4′,6‐diamidino‐2‐phenylindole dihydrochloride (DAPI; blue) and laminin (red) to indicate nuclei and myofibres, respectively, in extensor digitorum longus (EDL) muscles of P21 *Smn* ^*2B/+*^ and *Smn* ^*2B/−*^mice. Scale bars = 50 μm. Graphical summaries of (D) minimum ferret diameter and (E) shape factor index of myofibres from the EDL of *Smn* ^*2B/+*^ and *Smn* ^*2B/−*^ mice. *n* = 3. (F) mRNA content of several denervation markers including *cholinergic receptor nicotinic γ subunit* (*Chrnγ*), *neural cell adhesion molecule* (*Ncam*), *Myogenin* (*Myog*) and *RUNX family transcription factor 1* (*Runx1*) in the tibialis anterior (TA) muscles of *Smn* ^*2B/+*^ and *Smn* ^*2B/−*^ animals. *n* = 7–8. Data are expressed relative to the P9‐*Smn* ^*2B/+*^ group and are means ± SEM with individual data points displayed. #*p* < 0.05 vs. P9 within the same genotype, *p < 0.05 vs. P13 within the same genotype and ‡*p* < 0.05 between genotypes at the same timepoint, two‐way ANOVA.

We first sought to define disease‐specific changes in skeletal muscle size and innervation in *Smn* ^*2B/−*^ mice, which can influence mitochondrial and autophagic signalling. To this end, we collected skeletal muscle samples from *Smn* ^*2B/+*^ and *Smn* ^*2B/−*^ mice at P9, P13 and P21 to capture the pre‐symptomatic, early symptomatic and late symptomatic stages (Figure 1A). We observed notable atrophy in the TRI and EDL muscles of P21‐*Smn* ^*2B/−*^ mice relative to P21‐*Smn* ^*2B/+*^ as demonstrated by significantly lower muscle mass and myofibre minimum ferret diameter, respectively (Figure 1B–D). Furthermore, EDL myofibre morphology, assessed via shape factor index [S6], was significantly altered with increasing disease severity, which was concomitant with a dramatic upregulation of several transcriptional indicators of denervation (i.e., *Chrnγ*, *Ncam*, *Myog* and *Runx1*) in the TA muscles of late symptomatic SMA mice relative to age‐matched *Smn* ^*2B/+*^ animals (Figure 1E,F).

### 3.2 Mitochondrial Protein Expression Is Unchanged in Skeletal Muscle of Smn^2B/−^ Mice Despite Enhanced Signalling and Gene Expression

**FIGURE 2:**
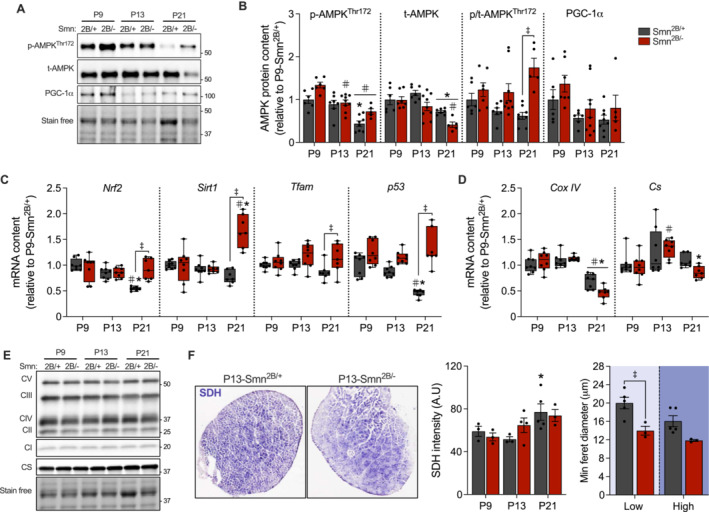
Mitochondrial protein content is unaffected by SMA disease progression. (A; left) Representative western blots of phosphorylated AMP‐activated protein kinase (p‐AMPK^Thr172^), total AMPK (t‐AMPK) and peroxisome proliferator‐activated receptor γ coactivator‐1α (PGC‐1α) protein levels in the TRI muscles of *Smn* ^*2B/+*^ and *Smn* ^*2B/−*^ mice. A stain‐free blot displayed below shows sample loading. Approximate molecular weights (kDa) shown at right of blots. (B) Graphical summaries of p‐AMPK^Thr172^, t‐AMPK, the p/t‐AMPK^Thr172^ ratio and PGC‐1α. *n* = 5–9. mRNA content of (C) *nuclear respiratory factor 2* (*Nrf2*), *sirtuin 1* (*Sirt1*), *mitochondrial transcript factor A* (*Tfam*) and *tumour protein 53* (*p53*), as well as (D) *cytochrome c oxidase subunit 4* (*Cox IV*) and *citrate synthase* (*Cs*) in the TA muscles of *Smn* ^*2B/+*^ and *Smn* ^*2B/−*^ animals. *n* = 7–8. (E) Typical western blots of subunits of mitochondrial oxidative phosphorylation (OxPhos) complexes I–V (CI–V) and CS. A stain‐free blot displayed below shows sample loading. Approximate molecular weights (kDa) shown at right of blots. *n* = 5–9. (F; left) Succinate dehydrogenase (SDH) staining of EDL muscles from *Smn* ^*2B/+*^ and *Smn* ^*2B/−*^ mice at P13. Graphs of mean SDH intensity in EDL of *Smn* ^*2B/+*^ and *Smn* ^*2B/−*^ (F; middle) and minimum ferret diameter of low (pale blue background) and high (dark blue background) SDH expressing fibres at P21 timepoint (F; right). (B‐D) Data are expressed relative to the P9‐*Smn* ^*2B/+*^ group and are means ± SEM with individual data points displayed. #*p* < 0.05 vs. P9 within the same genotype, *p < 0.05 vs. P13 within the same genotype and ‡*p* < 0.05 between genotypes at the same timepoint, two‐way ANOVA.

Next, we assessed the influence of SMA disease progression on molecules that regulate mitochondrial biogenesis as well as examined changes in organelle protein expression over time. Expansion of the mitochondrial reticulum is initiated, in part, by the activation of AMP‐activated protein kinase (AMPK) and its downstream target peroxisome proliferator–activated receptor ɣ coactivator‐1α (PGC‐1α) [9]. We observed an age‐dependent decline (*p* < 0.05) in phosphorylated (p‐AMPK^Thr172^) and total AMPK (t‐AMPK) protein content in the TRI muscles of *Smn* ^*2B/+*^ and *Smn* ^*2B/−*^ mice (Figure 2A,B). Interestingly, AMPK activation, defined by the ratio of p‐AMPK^Thr172^ to t‐AMPK, was ~2.8‐fold greater (*p* < 0.05) in P21‐*Smn* ^*2B/−*^ relative to *Smn* ^*2B/+*^ mice (Figure 2B). PGC‐1α protein levels did not differ between groups (Figure 2B). Next, we surveyed the mRNA expression in TA muscles of several genes important for the transcription of nuclear (nDNA) and mitochondrial (mtDNA) DNA‐encoded mitochondrial genes including *nuclear respiratory factor 2* (*Nrf2*), *sirtuin 1* (*Sirt1*), *mitochondrial transcript factor A* (*Tfam*) and *tumour protein 53* (*p53*). We noted an age‐dependent decline (*p* < 0.05) in *Nrf2*, *Tfam* and *p53* in the TA of *Smn* ^*2B/+*^ mice, such that all these biogenesis‐related transcripts were significantly higher in P21‐*Smn* ^*2B/−*^ animals compared to their *Smn* ^*2B/+*^ counterparts (Figure 2C). In contrast, genes that are indicative of mitochondrial content such as *cytochrome c oxidase subunit 4* (*Cox IV*) and *citrate synthase* (*Cs*) were either downregulated (*p* < 0.05) or unchanged in *Smn* ^*2B/−*^ mice (Figure 2D). Moreover, oxidative phosphorylation (OxPhos) complexes I–V (CI–V) and CS protein content in the TRI, total mitochondrial area in the TA, and succinate dehydrogenase (SDH) staining intensity in the EDL muscles were all similar between genotypes across all age groups (Figures 2E,F and S1A,B). Others have demonstrated that a subpopulation of ⍺‐motoneurons that are resistant to degeneration in pre‐clinical models of SMA are rich in mitochondria [18, S7], but it is unknown if this protection extends to the innervating muscle fibres. As such, we evaluated the degree of muscle wasting in low and high SDH fibres of P21 animals. Despite a considerable loss in the size of both low (*p* < 0.05) and high (*p* = 0.056) SDH fibres of the EDL muscles from *Smn* ^*2B/−*^ mice relative to healthy counterparts, we observed a minor sparing effect in myofibres with a greater abundance of SDH (−35%) compared to low expressing ones (−43%; Figures 2F and S1C).

### 3.3 Skeletal Muscle From Late Symptomatic Smn^2B/−^ Mice Exhibits an Accumulation of Autophagic and Mitophagic Markers

**FIGURE 3:**
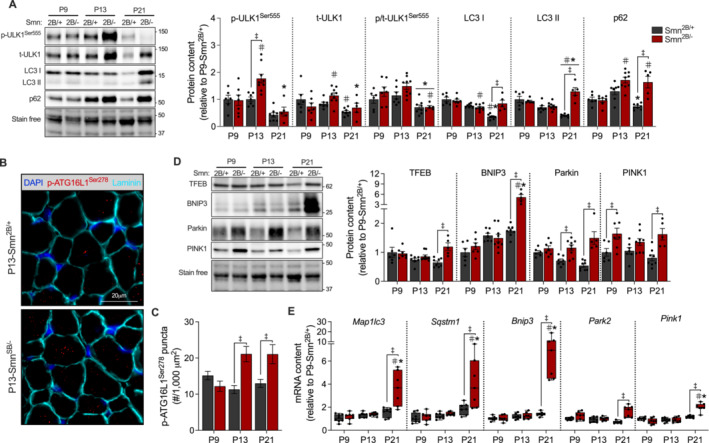
Enhanced autophagic and mitophagic signalling in skeletal muscle of symptomatic *Smn* ^*2B/−*^ mice. (A; left) Representative western blots of phosphorylated unc‐51‐like autophagy activating kinase 1 (p‐ULK1^Ser555^), total ULK1 (t‐ULK1), microtubule‐associated protein 1A/1B light chain 3 I (LC3 I), LC3 II and squestosome‐1 (p62) protein levels in the TRI muscles of *Smn* ^*2B/+*^ and *Smn* ^*2B/−*^ mice. A stain‐free blot displayed below shows sample loading. Approximate molecular weights (kDa) are displayed on each blot. (A; right) Graphical summaries of p‐ULK1^Ser555^, t‐ULK1, the p/t‐ULK1^Ser555^ ratio, LC3 I, LC3 II and p62 protein expression. *n* = 5–9. (B) Immunofluorescence microscopy of phosphorylated autophagy‐related 16‐like 1 (p‐ATG16L1^Ser278^) puncta (red), DAPI (blue) and laminin (cyan) to indicate newly forming autophagosomes, nuclei and myofibres, respectively, in EDL muscles of P13 *Smn* ^*2B/+*^ and *Smn* ^*2B/−*^mice. Scale bars = 20 μm. (C) Graphical summary of p‐ATG16L1^Ser278^ puncta per 1000 μm^2^. An average of 6 ± 2 field of views (FOVs) at 60× magnification were analysed for 4 biologically unique samples per group. *n* = 20–32 FOVs per group. (D; left) Representative western blots of transcription factor EB (TFEB), BCL2 interacting protein 3 (BNIP3), Parkin and PTEN‐induced kinase 1 (PINK1) protein expression. A stain‐free blot displayed below shows sample loading. Approximate molecular weights (kDa) shown at right of blots. (D; right) Graphical summary of TFEB, BNIP3, Parkin and PINK1 protein levels. *n* = 5–9. (E) mRNA levels of *Map 1lc3*, *Sqstm1*, *Bnip3*, *Park2* and *Pink1* in the TA muscles. *n* = 7–8. Data are expressed relative to the P9‐*Smn* ^*2B/+*^ group and are means ± SEM with individual data points displayed. #*p* < 0.05 vs. P9 within the same genotype, *p < 0.05 vs. P13 within the same genotype and ‡*p* < 0.05 between genotypes at the same timepoint, two‐way ANOVA.

Clearance of dysfunctional mitochondria (i.e., mitophagy) requires the initiation of autophagic processes to assemble mature autophagosomes, as well as the coordinated tagging of damaged organelles for removal [19, 20]. Therefore, we sought to investigate autophagy‐ and mitophagy‐related signalling in the skeletal muscle of *Smn* ^*2B/−*^ mice, as impairments in these processes may result in a suboptimal pool of mitochondria. The phosphorylation of the autophagy master switch, unc‐51‐like autophagy activating kinase 1 (p‐ULK1^Ser555^), was significantly higher in the TRI of P13‐*Smn* ^*2B/−*^ mice compared to P13‐*Smn* ^*2B/+*^ animals, but there were no genotype differences in the total expression of ULK1 (t‐ULK1) or its phosphorylation status (Figure 3A). Furthermore, microtubule‐associated protein 1A/1B light chain 3 I (LC3 I), LC3 II, LC3 II to I ratio and squestosome‐1 (p62) were 1.4–3.3‐fold greater (*p* < 0.05) in skeletal muscle of P21‐*Smn* ^*2B/−*^ mice in comparison to *Smn* ^*2B/+*^ (Figures 3A and S2A). These observations were concomitant with an upregulation of newly forming autophagosomes in EDL muscles of *Smn* ^*2B/−*^ mice at P13 and P21 as indicated by a significantly greater number of phosphorylated autophagy related 16‐like 1 (p‐ATG16L1^Ser278^) puncta, a well‐documented substrate of the autophagy mediating kinase, ULK1 (Figure 3B,C) [21]. Transcription factor EB (TFEB), an important upstream modifier of autophagic and lysosomal genes, was augmented (*p* < 0.05) in P21 *Smn* ^*2B/−*^ mice along with *Maplc3* and *Sqstm1* mRNA expression, which are transcriptional targets of TFEB (Figure 3D,E). In line with these findings, the expression of several key players involved in organelle degradation such as BCL2 interacting protein 3 (BNIP3), Parkin and PTEN‐induced kinase 1 (PINK1) were significantly increased at the protein and mRNA levels during late symptomatic stages of the disease (Figure 3D,E).

### 3.4 Enhanced Skeletal Muscle Mitochondrial Fission Markers and Fragmentation in Smn^2B/−^ Mice With Advanced Disease Progression

**FIGURE 4:**
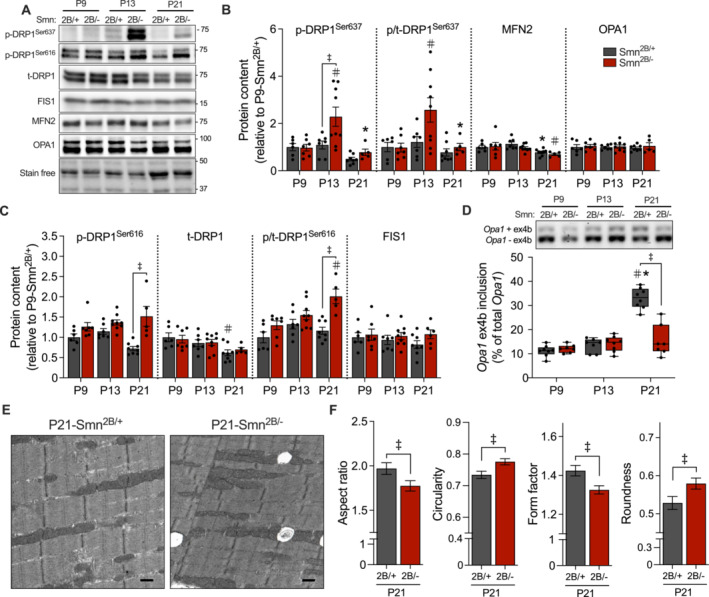
Dysregulated mitochondrial fission signalling results in a fragmented organelle phenotype. (A) Representative western blots of phosphorylated dynamics related protein 1 (DRP1) on serine 637 (p‐DRP1^Ser637^), serine 616 (p‐DRP1^Ser616^), total DRP1 (t‐DRP1), mitochondrial fission 1 (FIS1), mitofusin 2 (MFN2) and optic atrophy 1 (OPA1) protein levels in TRI muscles of *Smn* ^*2B/+*^ and *Smn* ^*2B/−*^ mice. A stain‐free blot displayed below shows sample loading. Approximate molecular weights (kDa) shown at right of blots. Graphical summaries of (B) p‐DRP1^Ser637^, the p/t‐DRP1^Ser637^ ratio, MFN2 and OPA1, as well as (C) p‐DRP1^Ser616^, t‐DRP1, the p/t‐DRP1^Ser616^ ratio and FIS1 protein expression. *n* = 5–9. (D) Representative end‐point PCR gel for *Opa1 +/−* exon (ex) 4b and a graphical summary of percent ex4b inclusion. *n* = 7–8. Data are expressed as means ± SEM with individual data points displayed. #*p* < 0.05 vs. P9 within the same genotype, *p < 0.05 vs. P13 within the same genotype and ‡*p* < 0.05 between genotypes at the same timepoint, two‐way ANOVA. (E) Transmission electron micrographs of intermyofibrillar mitochondria in the TA muscles of P21 *Smn* ^*2B/+*^ and *Smn* ^*2B/−*^ previously captured at 20 000× magnification. Scale bar = 0.5 μm. (F) Quantification of mitochondrial morphology parameters including aspect ratio, form factor, circularity and roundness. An average of 8 ± 2 FOVs at 15 000× magnification were analysed for 3 biologically unique samples per group. *n* = 21–29 FOVs per group. ‡*p* < 0.05 between genotypes at the same timepoint, two‐tail independent Student's *t*‐test for genotype comparison.

A continuous balance between organelle fusion and fission influences the structure and function of the mitochondrial network [22]. As such, we characterized the abundance of fusion‐ and fission‐related markers during various stages of disease severity. We observed a differential regulation of skeletal muscle dynamics related protein 1 (DRP1) throughout SMA progression. More specifically, DRP1 phosphorylation on the inhibitory serine 637 (p‐DRP1^Ser637^) site was augmented (*p* < 0.05) in TRI muscles of P13 *Smn* ^*2B/−*^ mice relative to *Smn* ^*2B/+*^, although its activation via the phosphorylation of serine 616 (p‐DRP1^Ser616^) was significantly elevated at P21 only (Figure 4A–C). In addition, protein levels of total DRP1 (t‐DRP1) and mitochondrial fission 1 (FIS1) were unchanged between genotypes across all timepoints, as were fusion‐related proteins mitofusin 2 (MFN2) and optic atrophy 1 (OPA1; Figure 4B,C). At the mRNA level, *Fis1* and *Mfn2* were more abundant (*p* < 0.05) in the TA muscles of P21 and P13 *Smn* ^*2B/−*^ animals, respectively, relative to their age‐matched *Smn* ^*2B/+*^ counterparts, but *Dnml1* and *Opa1* transcripts were similarly expressed (Figure S2B). Alternative splicing of *Opa1* ex4b, a key mediator of mtDNA transcription, was significantly blunted in late symptomatic *Smn* ^*2B/−*^ mice relative to *Smn* ^*2B/+*^ (Figure 4D). To gain a better understanding of mitochondrial structure in SMA‐like skeletal muscle, we performed a comprehensive quantitative assessment of organelle morphology using previously published electron micrographs from TA muscles of P21‐*Smn* ^*2B/+*^ and P21‐*Smn* ^*2B/−*^ mice [17]. Skeletal muscle mitochondria from *Smn* ^*2B/−*^ mice exhibited a lower (*p* < 0.05) aspect ratio (i.e., elongation) and form factor (i.e., branching) as well as displayed greater (*p* < 0.05) degrees of circularity and roundness, as compared to their *Smn* ^*2B/+*^ littermates (Figure 4E,F).

### 3.5 A Single Dose of Exercise Increases Mitochondrial Transcript Levels in Smn^2B/−^ Skeletal Muscle

**FIGURE 5:**
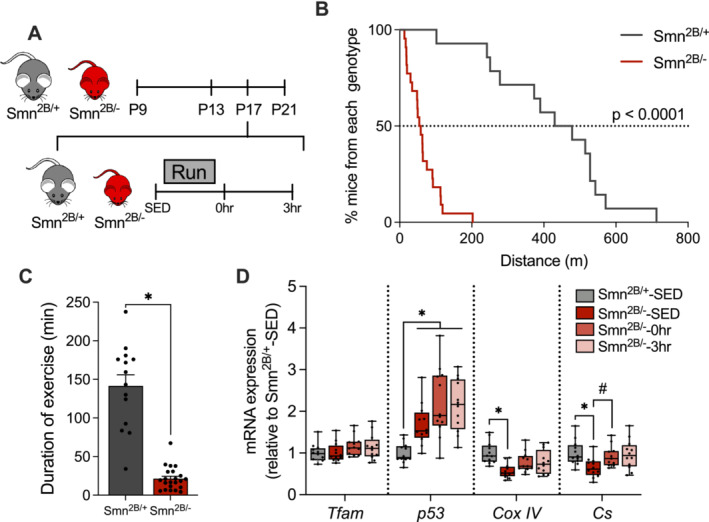
A single dose of exercise elicits the transcription of mitochondrial genes in *Smn* ^*2B/−*^ skeletal despite a blunted endurance capacity. (A) Schematic of the experimental design. Tissues were collected from *Smn* ^*2B/+*^ and *Smn* ^*2B/−*^ mice under sedentary (SED) conditions, immediately after (0 h) a single bout of exercise, as well as 3‐h (3 h) post‐exercise. (B) Kaplan–Meier analysis of the maximum distance travelled by *Smn* ^*2B/+*^ and *Smn* ^*2B/−*^ mice during treadmill running. (C) Duration of exercise on the motorized treadmill. *n* = 14–22. *p < 0.05 between genotypes at the same timepoint, two‐tail independent Student's *t*‐test for genotype comparison. (D) mRNA content of *Tfam*, *p53*, *Cox IV* and *Cs* in TA muscles of SED *Smn* ^*2B/+*^ mice, as well as *Smn* ^*2B/−*^ animals under SED, 0‐h and 3‐h conditions. *n* = 11–12. Data are expressed relative to the *Smn* ^*2B/+*^‐SED group and are means ± SEM with individual data points displayed. *p < 0.05 vs. WT‐SED and #*p* < 0.05 vs. *Smn* ^*2B/−*^‐SED, one‐way ANOVA.

Next, we utilized techniques to stimulate mitochondrial remodelling to potentially improve the dysregulated signalling observed in *Smn* ^*2B/−*^ skeletal muscle. Endurance‐type exercise is a potent stimulator of mitochondrial remodelling and has demonstrated benefits in SMA mice and patients [16, 23, 24]. Thus, we first aimed to investigate the molecular consequences of an acute bout of treadmill running on mitochondrial turnover and dynamics in skeletal muscle of *Smn* ^*2B/−*^ mice. Muscle samples were obtained from P17 *Smn* ^*2B/+*^ and *Smn* ^*2B/−*^ mice under resting and sedentary (SED) conditions (i.e., *Smn* ^*2B/+*^‐SED and *Smn* ^*2B/−*^‐SED), as well as from SMA‐like animals immediately after (*Smn* ^*2B/−*^‐0h) and 3‐h post‐exercise (*Smn* ^*2B/−*^‐3h; Figure 5A). Expectedly, *Smn* ^*2B/−*^ mice demonstrated a severe deficit in endurance capacity as evident by a significantly lower total distance travelled and duration of exercise (Figure 5B,C). Despite a lower tolerance for running, a single dose of exercise stimulated mitochondrial transcripts *Cox IV* (*p* = 0.1) and *Cs* (*p* < 0.05) in the TA muscles of *Smn* ^*2B/−*^‐0h mice but did not affect the expression of upstream regulators *Tfam* and *p53* (Figure 5D).

### 3.6 Exercise Blunts Autophagosome Formation and Enhances the LC3 II to I Ratio in Smn^2B/−^ Skeletal Muscle

**FIGURE 6:**
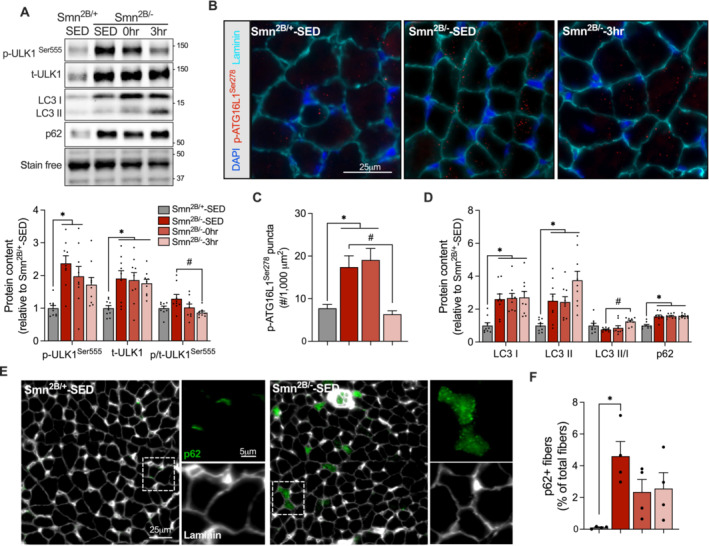
Altered autophagosome formation and maturation in SMA skeletal muscle following exercise. (A; top) Typical western blots of p‐ULK1^Ser555^, t‐ULK1, LC3 I, LC3 II and p62 protein levels in the TRI muscles of *Smn* ^*2B/+*^‐SED, *Smn* ^*2B/−*^‐SED, *Smn* ^*2B/−*^‐0h and *Smn* ^*2B/−*^‐3h animals. A stain‐free blot displayed below shows sample loading. Approximate molecular weights (kDa) shown at right of blots. (A; bottom) Graphical summaries of p‐ULK1^Ser555^, t‐ULK1 and ULK1 phosphorylation status, represented as the ratio of p‐ULK1^Ser555^/t‐ULK1. *n* = 8–9. (B) Immunofluorescence microscopy of p‐ATG16L1^Ser278^ puncta (red), DAPI (blue) and laminin (cyan) to indicate newly forming autophagosomes, nuclei and myofibres, respectively, in EDL muscles of mice from *Smn* ^*2B/+*^‐SED, *Smn* ^*2B/−*^‐SED and *Smn* ^*2B/−*^‐3h groups. Scale bar = 25 μm. (C) Graphical summary of p‐ATG16L1^Ser278^ puncta per 1000 μm^2^. An average of 6 ± 2 FOVs at 60× magnification were analysed from 4 biologically unique samples per group. *n* = 19–30 FOVs per group. (D) Graphical summaries of LC3 I, LC3 II, LC3 II/I ratio and p62 protein expression. *n* = 8–9. (E) Representative immunofluorescence staining for p62 (green) and laminin (white) in EDL muscles from *Smn* ^*2B/+*^‐SED and *Smn* ^*2B/−*^‐SED animals. Scale bar = 25 μm for lower magnification images and 5 μm for insets. (F) Quantification of myofibres positive for cytosolic p62 staining expressed as a percentage relative to total fibres. *n* = 3–4. Data are expressed relative to the *Smn* ^*2B/+*^‐SED group and are means ± SEM with individual data points displayed. *p < 0.05 vs. *Smn* ^*2B/+*^‐SED and #*p* < 0.05 vs. *Smn* ^*2B/−*^‐SED, one‐way ANOVA.

ULK1 stimulation is a crucial step in exercise‐induced mitophagy [19, 25]. Interestingly, we observed a significant decline in ULK1 phosphorylation status, defined by the ratio of p‐ULK1^Ser555^ to t‐ULK1, in TRI muscles of mice in the *Smn* ^*2B/−*^‐3h group relative to *Smn* ^*2B/−*^‐SED animals (Figure 6A). There was also a reduction (*p* < 0.05) in the number of p‐ATG16L1^Ser278^ puncta in the EDL muscles of *Smn* ^*2B/−*^‐3h compared to *Smn* ^*2B/−*^‐SED mice (Figure 6B,C). The LC3 II to I ratio was augmented (*p* < 0.05) 3‐h post‐exercise in *Smn* ^*2B/−*^ mice, whereas p62 protein content was unaltered with exercise (Figure 6D). We employed immunofluorescence staining for p62 in EDL cross‐sections to assess its localization and accumulation within myofibres. There was a significantly greater number of myofibres positive for cytoplasmic p62 protein in *Smn* ^*2B/−*^‐SED (4.6%) relative to *Smn* ^*2B/+*^‐SED (0.1%; Figure 6E,F). We observed only 2%–2.5% p62+ fibres in samples from *Smn* ^*2B/−*^‐0h and *Smn* ^*2B/−*^‐3h mice (Figure 6F). Using serial EDL cross‐sections, we did not observe any changes in the number of p‐ATG16L1^Ser278^ puncta in p62+ fibres compared to neighbouring p62− fibres (Figure S3A). Short‐term physical activity did not alter the abundance of mitophagy‐related proteins in *Smn* ^*2B/−*^ mice (Figure S3B,C) but did increase (*p* < 0.05) TA expression of *Park2* and *Pink1* relative to *Smn* ^*2B/−*^‐SED and *Smn* ^*2B/−*^‐SED, respectively (Figure S3D).

### 3.7 Acute Exercise Preferentially Inhibits DRP1 and Enhances Fusion Transcripts in Smn^2B/−^ Skeletal Muscle

**FIGURE 7:**
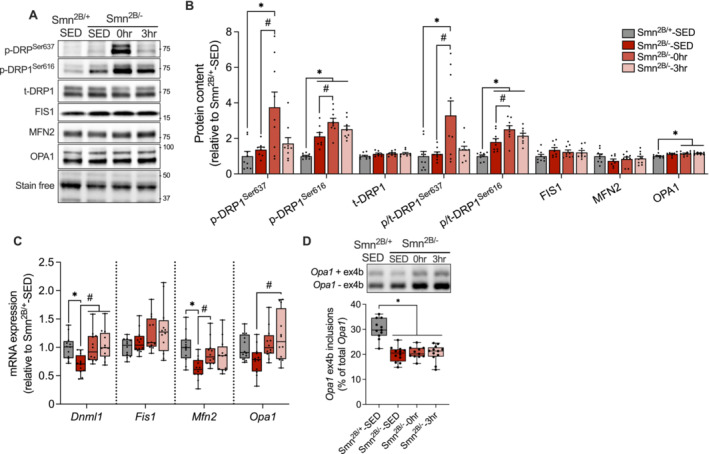
Enhanced exercise‐induced mitochondrial dynamics signalling in *Smn* ^*2B/−*^ mice. (A) Representative western blots of p‐DRP1^Ser637^, p‐DRP1^Ser616^, t‐DRP1, FIS1, MFN2 and OPA1 protein levels in TRI muscles from animals in the *Smn* ^*2B/+*^‐SED, *Smn* ^*2B/−*^‐SED, *Smn* ^*2B/−*^‐0h and *Smn* ^*2B/−*^‐3h cohorts. A stain‐free blot displayed below shows sample loading. Approximate molecular weights (kDa) shown at right of blots. (B) Graphical summaries of p‐DRP1^Ser637^, p‐DRP1^Ser616^, t‐DRP1 and DRP1 inhibition status, represented as the ratio of p‐DRP1^Ser637^/t‐DRP1 and DRP1 activation status, represented as the ratio of p‐DRP1^Ser616^/t‐DRP1, FIS1, MFN2 and OPA1 protein expression. *n* = 8–9. (C) mRNA content of *Dnml1*, *Fis1*, *Mfn2* and *Opa1* in TA samples of WT and SMA mice. *n* = 11–12. (D) Representative gel of end‐point PCR for *Opa1 +/−* ex4b and a graphical summary of percent ex4b inclusion. *n* = 11–12. Data are expressed relative to the *Smn* ^*2B/+*^‐SED group and are means ± SEM with individual data points displayed. *p < 0.05 vs. *Smn* ^*2B/+*^‐SED and #*p* < 0.05 vs. *Smn* ^*2B/−*^‐SED, one‐way ANOVA.

Given the aberrant mitochondrial phenotype in skeletal muscle of late symptomatic *Smn* ^*2B/−*^ mice (Figure 4), we examined the influence of acute exercise on organelle fusion and fission signalling. Running resulted in a robust, rapid and significant upregulation of p‐DRP1^Ser637^ and to a lesser extent p‐DRP1^Ser616^ (Figure 7A,B). This led to a 2.9‐ and 1.4‐fold increase (*p* < 0.05) in DRP1 inhibition status and DRP1 activation status, respectively, in the TRI muscles of *Smn* ^*2B/−*^‐0h animals relative to *Smn* ^*2B/−*^‐SED (Figure 7B). Treadmill running also augmented (p < 0.05) the expression of *Dnml1*, *Mfn2* and *Opa1*, but not *Fis1* (Figure 7C). Our group has previously demonstrated that acute physical activity augments the inclusion of *Opa1* ex4b in the skeletal muscle of *Smn* ^*2B/+*^ mice and other models of NMD [26]. Nevertheless, alternative splicing of *Opa1* ex4b was unchanged in response to a single dose of exercise (Figure 7D).

### 3.8 Stimulation of Autophagic and Mitophagic Pathways via UA Enhances Mitochondrial Content and Function in SMA Patient Muscle Cells

**FIGURE 8:**
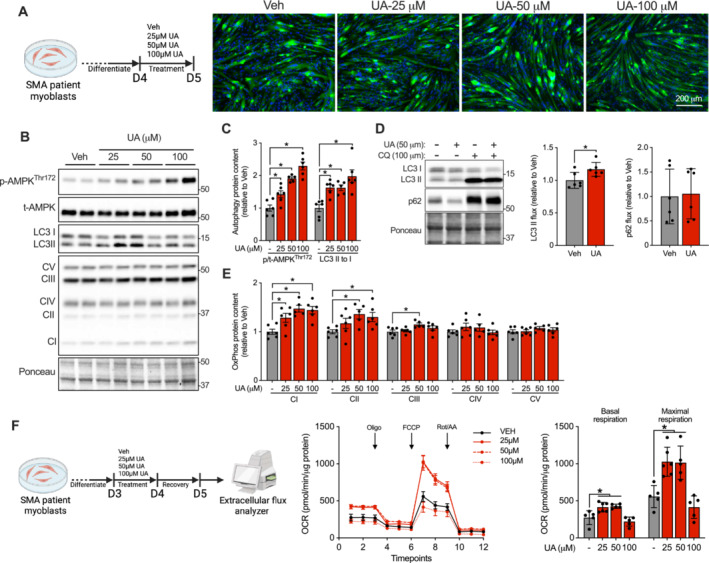
Urolithin A (UA) augments autophagy and mitophagy signalling as well as enhances mitochondrial content and function in SMA patient cells. (A; left) A schematic of the dosing strategy. Immortalized myoblasts isolated from a Type I SMA patient were differentiated for 4 days (D4), after which myotubes were dosed with either the dimethyl sulfoxide vehicle (Veh) or UA (25, 50 or 100 μM) for 24 h and collected for further processing at Day 5 (D5). (A; right) Representative immunofluorescence images of myosin heavy chain I (green) and DAPI (blue) staining in SMA patient myotubes treated with Veh or UA (25, 50 or 100 μM). (B) Typical western blots of p‐AMPK^Thr172^, t‐AMPK, LC3 I/II and OxPhos. A Ponceau S stain displayed below shows sample loading. Approximate molecular weights (kDa) shown at right of blots. Graphical summaries of (C) p/t‐AMPK^Thr172^ ratio and LC3 II to I. (D; left) Representative western blots of LC3 I/II and p62 in SMA patient‐derived myotubes treated for 24 h with Veh or 50 μM UA and for 12 h with 100 μM chloroquine (CQ) or the corresponding Veh. A Ponceau S stain displayed below shows sample loading. Approximate molecular weights (kDa) shown at right of blots. (D; right) Graphical summaries for LC3 II and p62 flux in Veh or UA‐treated myotubes. (E) OxPhos protein expression in Veh or UA‐treated SMA myotubes. (F; left) Visual schematic of the experimental design to measure oxygen consumption rates (OCR). SMA patient‐derived myotubes were treated with Veh or UA for a 24‐h period, after which cells were allowed to equilibrate in differentiation media for an additional 24 h before OCR were measured. (F; middle) Cellular respiration tracings in response to the addition of oligomycin (Oligo), FCCP, rotenone (Rot) and antimycin A (AA). (F; right) Summary of basal and maximal OCR in SMA myotubes normalized to total protein content. *n* = 5–6 technical replicates for each treatment condition. Data are expressed relative to Veh and are means ± SEM with individual data points displayed. *p < 0.05 vs. Veh, one‐way ANOVA for (B–D) and (F); two‐tail independent Student's *t*‐test for (E).

UA is a mitochondrial‐targeted agent that augments skeletal muscle health and function under normal and other myopathic conditions such as in Duchenne muscular dystrophy [15, 27]. Thus, immortalized skeletal muscles cells obtained from an 11‐year‐old SMA patient (0 *SMN1* and 3 *SMN2* copies) were differentiated for 4 days (D4) and then incubated with either the vehicle (Veh) or varying doses of UA (25, 50 or 100 μM) for a 24‐h period (Figure 8A). UA‐treated SMA myotubes did not demonstrate overt morphological differences or changes in expression of early myogenic activation marker myoblast differentiation protein 1 (MyoD) relative to Veh treated cells (Figures 8A and S4A,B). In contrast, a significant downregulation in myogenin, an important player in myogenic differentiation [28], was noted at all doses of UA treatment (Figure S4B). We observed a dose‐dependent increase (*p* < 0.05) in AMPK phosphorylation status with UA treatment (Figure 8B,C). Given that AMPK is a key regulator of autophagic signalling and mitochondrial biogenesis, we assessed markers of organelle degradation and mitochondrial content. The LC3 II to I ratio and total BNIP3 protein expression were significantly increased following myotube exposure to UA, but no changes were observed in the abundance of p62 (Figures 8C and S4C). To directly evaluate the ability of UA to stimulate autophagic flux, D4 SMA myotubes were treated with either Veh or UA (50 μM) for 24 h and were co‐incubated with either Veh or CQ (100 μM) for 12 h. UA induced a ~20% increase (*p* < 0.05) in LC3 II flux, although no change was observed with p62 (Figure 8D). Additionally, UA elicited a significant upregulation of mitochondrial OxPhos complexes I–III in patient derived myotubes (Figure 8E). To assess mitochondrial function, we treated D4‐differentiated SMA patient muscle cells with Veh or UA for 24 h and assessed oxygen consumption rates (OCR; Figure S4D). UA induced a significant blunting of basal and maximal cellular respiration at all doses relative to Veh (Figure S4E,F). Other indirect and direct AMPK activators and mitochondria‐simulating agents, such as metformin and O304, rapidly and transiently diminish mitochondrial OxPhos prior to subsequent enhancements in organelle function [S8, S9]. We hypothesized that UA may work via a similar mechanism. Thus, D3‐differentiated SMA myotubes were dosed with Veh or UA for 24 h, allowed to recover in DM for another 24 h, and then OCR were measured (Figure 8F). Lower doses of UA (25 and 50 μM) elicited ~50%–80% increases (*p* < 0.05) in basal and maximal OCR relative to untreated Veh myotubes (Figure 8F).

## 4 Discussion

In this study, we aimed to define skeletal muscle mitochondrial turnover, dynamics and mechanisms of macro‐autophagy during disease progression in a mouse model of SMA. Additionally, we investigated physiological and pharmacological approaches to stimulate organelle plasticity in SMA murine muscle in vivo and in patient myotubes in vitro, respectively. Our data demonstrate that skeletal muscle expression of mitochondrial protein markers such as OxPhos and CS and SDH intensity were similar between *Smn* ^*2B/+*^ and *Smn* ^*2B/−*^mice regardless of disease progression, due in part to enhanced compensatory upstream transcriptional activity of *Nrf2*, *Tfam* and *p53* in SMA‐like animals. Interestingly, we found a minor protective effect of muscle wasting in fibres with high amounts of SDH. Furthermore, symptomatic (i.e., pre‐atrophic) and late symptomatic (i.e., atrophic) *Smn* ^*2B/−*^ skeletal muscle was characterized by augmented autophagic and mitophagic markers and evidence of dysfunctional mitochondria, indicating impaired organelle degradation with increasing disease severity. We also observed aberrant organelle fusion and fission signalling, which was associated with abnormal splicing of *Opa1* transcripts and a fragmented mitochondrial morphology in late stages of SMA. Altogether, these data indicate that mitochondrial homeostasis is perturbed in *Smn* ^*2B/−*^ skeletal muscle due to dysregulated organelle recycling and enhance fission signalling, which occurs concomitantly with disease progression. Targeting mitochondrial remodelling through a single dose of exercise resulted in augmented mitochondrial transcripts and autophagic signalling in *Smn* ^*2B/−*^ mice. Moreover, in patient muscle cells, UA administration elicited AMPK activation, autophagic flux, mitochondrial biogenesis and mitophagy markers, as well as improved oxidative capacity. Collectively, our findings illustrate that skeletal muscle mitochondrial turnover and dynamics are concurrently and progressively impacted during the SMA disease course and support deploying physiological and pharmacological mitochondrial‐targeted strategies to effectively remodel organelle structure and function.

Mitochondrial content and function are known to be affected in several organs of pre‐clinical SMA models [S10, 29, 30]. However, data on the impact of SMA pathology on the abundance of skeletal muscle mitochondria are equivocal [9]. Our results revealed comparable levels of OxPhos proteins and SDH expression in skeletal muscle samples across the lifespan of a SMA mouse model relative to age‐matched healthy mice. This was surprising since denervation‐induced muscle atrophy, a prominent feature of SMA, has been shown to profoundly reduce mitochondrial content [S11–S13]. Interestingly, we noted that oxidative myofibres with high levels of SDH are partially protected against SMA‐associated muscle wasting. These findings are in line with previous literature showing that mitochondria‐rich ⍺‐motoneurons are resilient to SMA pathogenesis [18, S7]. We propose that the similarities in OxPhos expression between genotypes may be due, in part, to the enhanced expression of key transcriptional regulators of mitochondrial genes at the nDNA in SMA skeletal muscle, such as *Nrf2* and *Sirt1*, as well as at the mtDNA, including *Tfam* and *p53*. Furthermore, aberrant macro‐autophagy and mitophagy may also explain the analogous levels of mitochondrial protein abundance between *Smn* ^*2B/+*^ and *Smn* ^*2B/−*^ mice. Our data supporting this hypothesis indicate that although *Smn* ^*2B/−*^ skeletal muscle exhibited a greater capacity for autophagosome formation (i.e., higher p‐ATG16L1^Ser278^ puncta) and maturation (i.e., augmented LC3 II expression), the expression of mitophagy markers BNIP3, PINK1 and Parkin remain significantly increased in symptomatic, as well as in late symptomatic *Smn* ^*2B/−*^ mice, suggesting an irregularity in the clearance rate of damaged mitochondria. Although mitophagy‐related markers can be elevated due to both suboptimal or enhanced organelle degradation, recent work has demonstrated that skeletal muscle–specific deletion of SMN protein results in an accumulation of morphologically abnormal organelles and mitophagy‐specific proteins due to a downregulation in autophagic flux [31]. Thus, given that the processes required for the initiation of macro‐autophagy remain intact in skeletal muscle of *Smn* ^*2B/−*^ mice, a reasonable hypothesis is that the blunted recycling of dysregulated mitochondria may be attributed to impaired lysosomal biogenesis and/or fusion. This phenomenon has been previously observed in ageing skeletal muscle and other NMDs [32, 33, 34] and thus may also contribute to the SMA muscle phenotype. Finally, comparable levels of mitochondrial proteins may be explained by a blunted translational capacity concomitant with diminished organelle degradation. Although this was not investigated in the present study, others have demonstrated that the brain and spinal cord of SMA mice display dysregulated ribosomal biology and lower translational efficiency [S14]. A limitation of the present study is the lack of autophagic and mitophagic flux experiments in vivo. Perturbations in autophagy may indirectly influence mitochondrial health through impaired redox biology and abnormal proteostasis. As such, further investigations are required to decipher the interplay between autophagy and mitochondrial biology in SMA skeletal muscle. Altogether, the data indicate that the similarities in skeletal muscle mitochondrial abundance between *Smn* ^*2B/+*^ and *Smn* ^*2B/−*^ mice during disease progression are due to enhanced expression of upstream transcriptional mediators and diminished capacity for organelle degradation.

Optimal health of the mitochondrial reticulum is dependent on ongoing processes of fusion and fission (i.e., dynamics). An imbalance between these pathways in skeletal muscle results in mitochondrial dysfunction and several associated molecular perturbations resulting in accelerated atrophy [35]. Although SMA neuronal cells are known to display a simplistic mitochondrial network with low motility [18, 36, 37], the consequences of SMA disease progression on mechanisms regulating mitochondrial dynamics in skeletal muscle have not been investigated. Our results revealed that DRP1 phosphorylation, and thereby its activity, was dependent on SMA disease stages. For instance, greater phosphorylation at the serine 637 site, indicative of DRP1 inhibition, was observed in early symptomatic SMA mice before significant muscle atrophy has occurred, whereas a shift towards enhanced expression of the serine 616 activation mark was noted at P21 during the peak atrophic phenotype. In contrast, the expression of fusion proteins such as OPA1 and MFN2 remained stable as disease severity advanced. Notably, the inclusion of *Opa1* ex4b through alternative splicing was substantially impaired at P21, likely due to the critical involvement of SMN protein in spliceosome assembly and function [1]. In line with these findings, our ultrastructural analysis of mitochondrial morphology further supports the presence of a fragmented mitochondrial phenotype during the final stages of disease progression. Others have shown that skeletal muscle–specific overexpression of DRP1 blunts myofibre growth and negatively impacts muscle integrity in aged mice [38, 39]. Our group has previously demonstrated that skeletal muscle atrophy in *Smn* ^*2B/−*^ mice predominantly occurred between P13 and P21 [16]. As such, we suspect that DRP1 regulation of organelle morphology promotes skeletal muscle mitochondrial fragmentation in SMA, which partly contributes to muscle weakness and wasting during symptomatic stages of the disease. It is important to note that the present findings may be influenced by the denervation‐induced atrophy of myofibres in this model, which is also a hallmark characteristic of the natural progression of SMA. However, dysregulation of metabolic and mitochondrial processes has been observed in other tissues that are not impacted by ⍺‐motoneurons such as the liver [S10, S15]. Future work should investigate this hypothesis further by directly manipulating DRP1 activity in SMA.

Exercise is a safe and practical lifestyle intervention that elicits pleiotropic physiological and molecular benefits in individuals with less severe Type II–IV SMA [23, 40], a relative minority of SMA patients. However, thanks to remarkable, recent advances in small molecule and gene therapies [6], Type I SMA patients will experience less severe forms of the disorder and will therefore be amenable to exercise prescription to further enhance quality of life. Thus, our Type II–like mice represent a translatable model for exercise effects in SMA patients. In unaffected individuals, exercise‐induced physiological benefits are underpinned by remodelling of mitochondrial biology that ultimately leads to enhanced respiratory capacity as well as an elongated reticulum of healthy organelles [7]. In the current study, we demonstrate that a single dose of exercise to exhaustion stimulates the expression of mitochondrial transcripts in *Smn* ^*2B/−*^ skeletal muscle. Additionally, exercise normalized the pathological upregulation of ULK1 phosphorylation at the serine 555 mark as well as completely rescued indicators of excessive formation of autophagosomes (i.e., p‐ATG16L1^Ser278^ puncta). It is worth noting that these data contrast the observations in healthy animals, which demonstrate a rapid upregulation of p‐ULK1^Ser555^ and p‐ATG16L1^Ser278^ immediately after maximal exercise [19, 25, 26]. Despite the downregulation of autophagy initiation markers, we observed a significant increase in the LC3 II to I ratio and a non‐statistically significant decrease in myofibre accumulation of p62 protein, suggesting an enhanced capacity for degradation of damaged cellular machinery. We and others have highlighted that acute running elicits temporal regulation of DRP1 activity whereby p‐DRP1^Ser616^ peaks immediately after exercise followed by increased p‐DRP1^Ser637^ residues 3‐h post exercise [S16, 19, 26]. Interestingly, this time‐dependent regulation is lost in SMA skeletal muscle as acute physical activity stimulated simultaneous phosphorylation of DRP1 on the Ser 637 and 616 marks in *Smn* ^*2B/−*^ mice. Lastly, acute exercise restored the expression of fusion and fission mRNA molecules including *Dnml1*, *Mfn2* and *Opa1*. In contrast to previous evidence from our laboratory in healthy WT animals and mice with myotonic dystrophy [26], exercise did not influence alternative splicing of *Opa1* in *Smn* ^*2B/−*^ mice, which suggests that exercise therapy for skeletal muscle mitochondrial dysfunction depends on the pathological context. Of note, there was significant variability in the maximal exercise capacity of *Smn* ^*2B/−*^ mice, which can influence exercise‐induced molecular pathways and is therefore a limitation of the present study. Thus, a single dose of exercise alters autophagic and mitochondrial signalling in skeletal muscle of *Smn* ^*2B/−*^ mice to promote correct assembly of the autophagosome and activate signalling cascades that may lead to organelle remodelling. Although chronic exercise training elicits neuroprotective effects in pre‐clinical models of SMA [41], it is unlikely that these adaptations occurred acutely (i.e., 0–3‐h post‐exercise) and were observable in the present study.

UA is a naturally derived compound that is emerging as a potential therapy for conditions linked to mitochondrial deficits such as ageing and other diseases including NMDs [15]. Several lines of preclinical and clinical evidence attribute the UA‐induced improvements on skeletal muscle health to the cellular activation of autophagy and mitophagy, as well as to the upregulation of a healthy pool of mitochondria [27, 42]. We speculated that treatment of SMA patient‐derived skeletal muscle cells with UA would also result in these molecular and functional benefits. In line with previous data in human primary muscle cells [27], UA resulted in a remarkable decrease in myogenin expression in SMA myotubes that did not have any deleterious effects on myotube formation or differentiation, which suggests that these effects are temporary but require further investigations. UA exposure enhanced the activation of the upstream kinase, AMPK as well as stimulated the maturation of autophagosomes (i.e., enhanced LC3 II to I ratio), increased autophagic flux and augmented the expression of mitochondrial electron transport chain machinery. Greater abundance of OxPhos proteins translated into a significant improvement in mitochondrial respiration following a 24‐h recovery period but not immediately after UA, which suggests that UA acutely induces cellular stress to elicit chronic mitochondrial adaptations. This transient and temporal inhibition of mitochondrial oxidative capacity has been observed with the use of alternative AMPK activators such as metformin and O304 [S8, S9]. Collectively, our results suggest that UA is a promising intervention for targeting SMA‐associated skeletal muscle mitochondrial dysfunction through enhanced organelle turnover.

In summary, skeletal muscle mitochondrial biology and macro‐autophagy were responsive to SMA trajectory, such that their alterations were associated with accelerated skeletal muscle denervation and wasting as disease severity increased. Although there was stable expression of mitochondrial proteins between *Smn* ^*2B/+*^ and *Smn* ^*2B/−*^ mice during late symptomatic stages of the disorder, we observed in the SMA condition an upregulation of upstream transcriptional mediators crucial for organelle biogenesis along with mitochondria that displayed a fragmented molecular and morphological phenotype and an accumulation of proteins indicative of dysfunctional mitochondria due to aberrant autophagy and mitophagy. These findings indicate that despite compensatory attempts to maintain mitochondrial homeostasis, defective organelle turnover and dynamics are inherent characteristics of SMA pathogenesis in skeletal muscle. A single dose of exercise in *Smn* ^*2B/−*^ mice and UA treatment of SMA patient cells partially attenuated dysregulated organelle turnover and dynamics signalling in skeletal muscle as well as stimulated mitochondrial function in patient muscle cells. We conclude that abnormalities in skeletal muscle mitochondrial turnover and dynamics are apparent during SMA disease progression and that pharmacological and lifestyle‐based approaches are effective in stimulating therapeutic organelle plasticity.

## Conflicts of Interest

The authors declare no conflicts of interest.

## Supporting information


**Figure S1** Similar expression of OxPhos proteins and mitochondrial surface area in *Smn* ^*2B/+*^ and *Smn* ^*2B/−*^ mice. (A) Relative expression of mitochondrial oxidative phosphorylation CI–V and CS proteins in the TRI muscles of *Smn* ^*2B/+*^ and *Smn* ^*2B/−*^ mice. *n* = 5–9. Data are expressed relative to the P9‐*Smn* ^*2B/+*^ group and are means ± SEM with individual data points displayed. (B) Quantification of percent mitochondrial area relative to the total area of the FOV. An average of 8 ± 2 FOVs at 15 000× magnification were analysed for 3 biologically unique samples per group. *n* = 21–29 FOVs per group. (C) Mean SDH intensity of low (pale blue background) and high (dark blue background) SDH expressing fibres in the EDL muscles of P25 animals.
**Figure S2.** LC3 II to I ratio and transcriptional expression of key regulators of mitochondrial dynamics. (A) The ratio of LC3 II to I protein content in TRI muscles of *Smn* ^*2B/+*^ and *Smn* ^*2B/−*^ mice. *n* = 5–9. (B) mRNA expression of *Dnml1*, *Fis1*, *Mfn2* and *Opa1* in TA samples of *Smn* ^*2B/+*^ and *Smn* ^*2B/−*^ mice. *n* = 7–8. Data are expressed relative to the P9‐*Smn* ^*2B/+*^ group and are means ± SEM with individual data points displayed. #*p* < 0.05 vs. P9 within the same genotype, *p < 0.05 vs. P13 within the same genotype and ‡*p* < 0.05 between genotypes at the same timepoint, two‐way ANOVA.
**Figure S3.** Acute exercise does not alter the abundance of mitophagy‐related protein in skeletal muscle of SMA mice. (A) Immunofluorescence microscopy of (top panel) p62 (green) and laminin (white), as well as (bottom panel) p‐ATG16L1^Ser278^ puncta (red), DAPI (blue) and laminin (cyan) in EDL serial cross‐sections of mice from *Smn* ^*2B/−*^‐SED, and *Smn* ^*2B/−*^‐3h animals. White asterisk denotes p62 positive fibres and yellow asterisk identify neighbouring p62 negative fibres. (B) Western blots of TFEB, BNIP3, Parkin, and PINK1 protein expression. A stain‐free blot displayed below shows sample loading. Approximate molecular weights (kDa) shown at right of blots. (C) Graphical summaries of TFEB, BNIP3, Parkin and PINK1 protein content. *n* = 8–9. (D) mRNA levels of *Park2*, and *Pink1* in TA muscles of mice in the four experimental groups. *n* = 11–12. Data are expressed relative to the *Smn* ^*2B/+*^‐SED group and are means ± SEM with individual data points displayed. * *p* < 0.05 vs. WT‐SED, and # p < 0.05 vs. *Smn* ^*2B/−*^‐SED, one‐way ANOVA.
**Figure S4.** Myogenic protein expression and cellular respiration of SMA myotubes immediately after Veh or UA treatment (A) Typical Western blots of myoblast differentiation protein 1 (MyoD), myogenin, p62 and BNIP3 proteins in SMA patient‐derived myotubes following 24 h of Veh or UA administration. A Ponceau S stain displayed below shows sample loading. Approximate molecular weights (kDa) shown at right of blots. Graphical summaries of (B) myogenic proteins including MyoD, and myogenin as well as (C) autophagy‐related proteins such as p62 and BNIP3. (D) Visual schematic of the experimental design to measure OCR in SMA patient‐derived myotubes immediately after 24 h of Veh or UA treatment. (E) Cellular respiration tracings in response to the addition of Oligo, FCCP, Rot and AA. (F) Summary of basal and maximal OCR in SMA myotubes normalized to total protein content. *n* = 5–6 technical replicates for each treatment condition. Data are expressed relative to Veh and are means ± SEM with individual data points displayed. *p < 0.05 vs. Veh, one‐way ANOVA.
**Table S1.** List of primary antibodies.
**Table S2.** PCR primer sequences.
