# Supplementary material for: Skeletal Muscle Mitochondrial and Autophagic Dysregulation Are Modifiable in Spinal Muscular Atrophy
Source: J Cachexia Sarcopenia Muscle. 2025 Feb 3;16(1):e13701. doi: 10.1002/jcsm.13701 (PMC11790611; doi:10.1002/jcsm.13701)
Supplement: Supplementary file 1 — Figure S1 Similar expression of OxPhos proteins and mitochondrial surface area in Smn 2B/+ and Smn 2B/− mice. (A) Relative expression of mitochondrial oxidative phosphorylation CI–V and CS proteins in the TRI muscles of Smn 2B/+ and Smn 2B/− mice. n = 5–9. Data are expressed relative to the P9‐Smn 2B/+ group and are means ± SEM with individual data points displayed. (B) Quantification of percent mitochondrial area relative to the total area of the FOV. An average of 8 ± 2 FOVs at 15 000× magnification were analysed for 3 biologically unique samples per group. n = 21–29 FOVs per group. (C) Mean SDH intensity of low (pale blue background) and high (dark blue background) SDH expressing fibres in the EDL muscles of P25 animals. Figure S2. LC3 II to I ratio and transcriptional expression of key regulators of mitochondrial dynamics. (A) The ratio of LC3 II to I protein content in TRI muscles of Smn 2B/+ and Smn 2B/− mice. n = 5–9. (B) mRNA expression of Dnml1, Fis1, Mfn2 and Opa1 in TA samples of Smn 2B/+ and Smn 2B/− mice. n = 7–8. Data are expressed relative to the P9‐Smn 2B/+ group and are means ± SEM with individual data points displayed. #p < 0.05 vs. P9 within the same genotype, *p < 0.05 vs. P13 within the same genotype and ‡p < 0.05 between genotypes at the same timepoint, two‐way ANOVA. Figure S3. Acute exercise does not alter the abundance of mitophagy‐related protein in skeletal muscle of SMA mice. (A) Immunofluorescence microscopy of (top panel) p62 (green) and laminin (white), as well as (bottom panel) p‐ATG16L1Ser278 puncta (red), DAPI (blue) and laminin (cyan) in EDL serial cross‐sections of mice from Smn 2B/− ‐SED, and Smn 2B/− ‐3h animals. White asterisk denotes p62 positive fibres and yellow asterisk identify neighbouring p62 negative fibres. (B) Western blots of TFEB, BNIP3, Parkin, and PINK1 protein expression. A stain‐free blot displayed below shows sample loading. Approximate molecular weights (kDa) shown at right of blots. (C) Graphical summaries of [file JCSM-16-e13701-s001.docx]

**SUPPLEMENTARY MATERIAL**

**TITLE**: Skeletal muscle mitochondrial and autophagic dysregulation are modifiable in spinal muscular atrophy

**AUTHORS:** Andrew I Mikhail^1^, Sean Y Ng^1^, Donald Xhuti^2^, Magda A Lesinski^1^, Jennifer Chhor^1^, Marc-Olivier Deguise^3,4,5^, Yves De Repentigny^3^, Joshua P Nederveen^2^, Rashmi Kothary^3,6,7^, Mark A Tarnopolsky^2^, and Vladimir Ljubicic^1^*

**AFFILIATION:**

^1^Department of Kinesiology, McMaster University, Hamilton, Ontario, Canada

^2^Department of Pediatrics, McMaster University Medical Center, Hamilton, Ontario, Canada

^3^Regenerative Medicine Program, Ottawa Hospital Research Institute, Ottawa, Ontario, Canada

^4^Faculty of Medicine, University of Ottawa, Ottawa, Ontario, Canada

^5^Division of Neonatology, Department of Pediatrics, Children’s Hospital of Eastern Ontario, Ottawa, Ontario, Canada

^6^Department of Medicine, University of Ottawa, Ottawa, Ontario, Canada

^7^Department of Cellular and Molecular Medicine, University of Ottawa, Ottawa, K1H 8M5, Canada and Centre for Neuromuscular Disease, University of Ottawa, K1H 8M5, Canada

**SUPPLEMENTARY FIGURES**

**
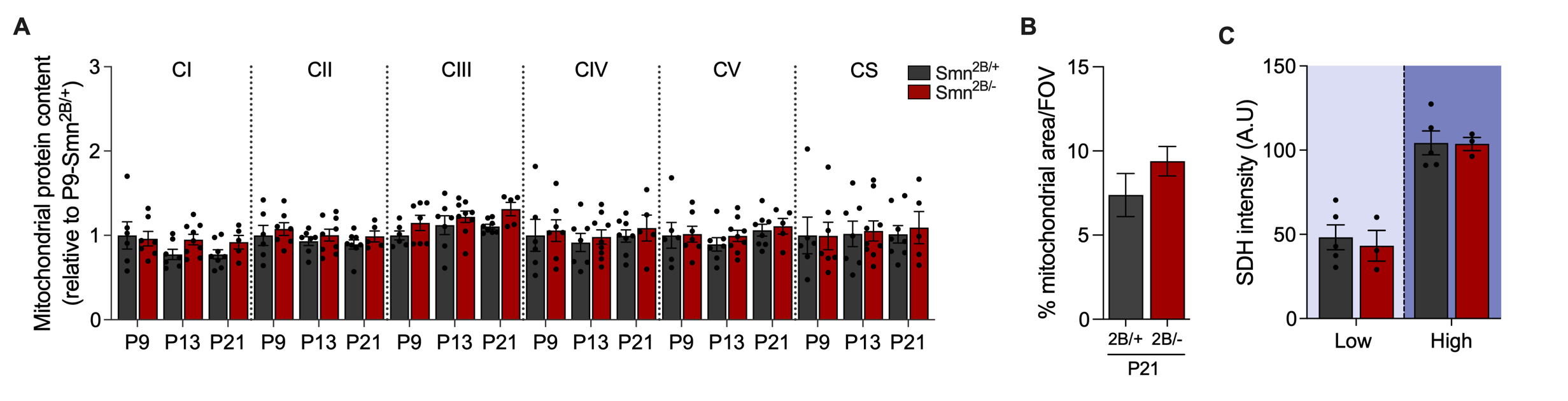
**

**Figure S1. Similar expression of OxPhos proteins and mitochondrial surface area in *Smn^2B/+^* and *Smn^2B/-^* mice. (A)** Relative expression of mitochondrial oxidative phosphorylation CI–V, and CS proteins in the TRI muscles of *Smn^2B/+^* and *Smn^2B/-^* mice. n = 5-9. Data are expressed relative to the P9-*Smn^2B/+^* group and are means ± SEM with individual data points displayed. **(B)** Quantification of percent mitochondrial area relative to the total area of the FOV. An average of 8 ± 2 FOVs at 15,000x magnification were analyzed for 3 biologically unique samples per group. n = 21-29 FOVs per group. **(C)** Mean SDH intensity of low (pale blue background) and high (dark blue background) SDH expressing fibers in the EDL muscles of P25 animals.

**
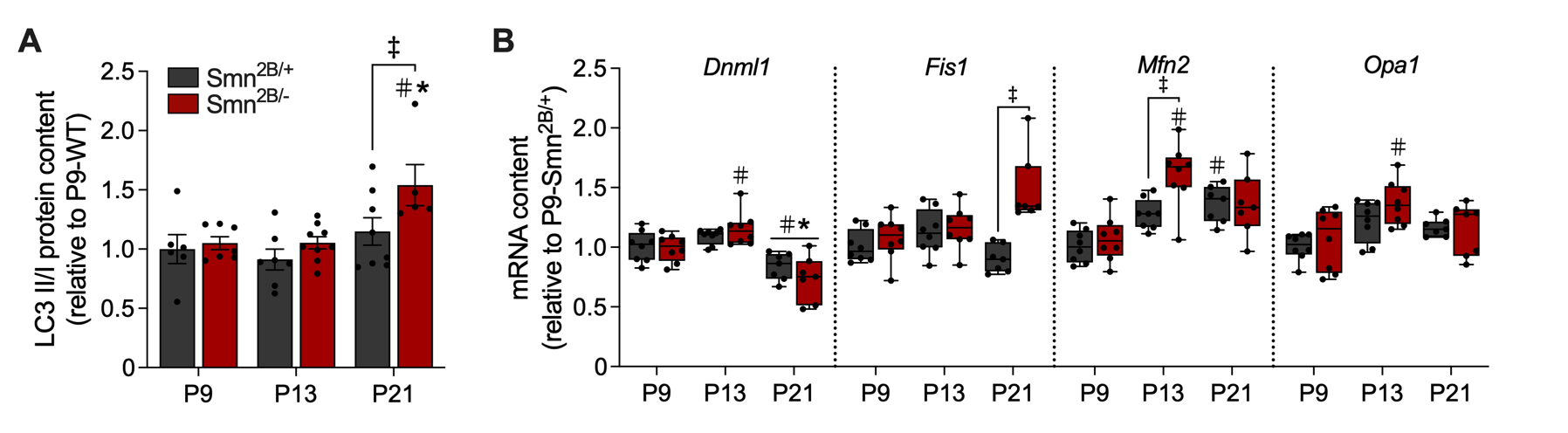
**

**Figure S2. LC3 II to I ratio and transcriptional expression of key regulators of mitochondrial dynamics. (A)** The ratio of LC3 II to I protein content in TRI muscles of *Smn^2B/+^* and *Smn^2B/-^* mice. n = 5-9. **(B)** mRNA expression of *Dnml1*, *Fis1*, *Mfn2*, and *Opa1* in TA samples of *Smn^2B/+^* and *Smn^2B/-^* mice. n = 7-8. Data are expressed relative to the P9-*Smn^2B/+^* group and are means ± SEM with individual data points displayed. # p < 0.05 vs. P9 within the same genotype, * p < 0.05 vs. P13 within the same genotype, and ‡ p < 0.05 between genotypes at the same timepoint, two-way ANOVA.


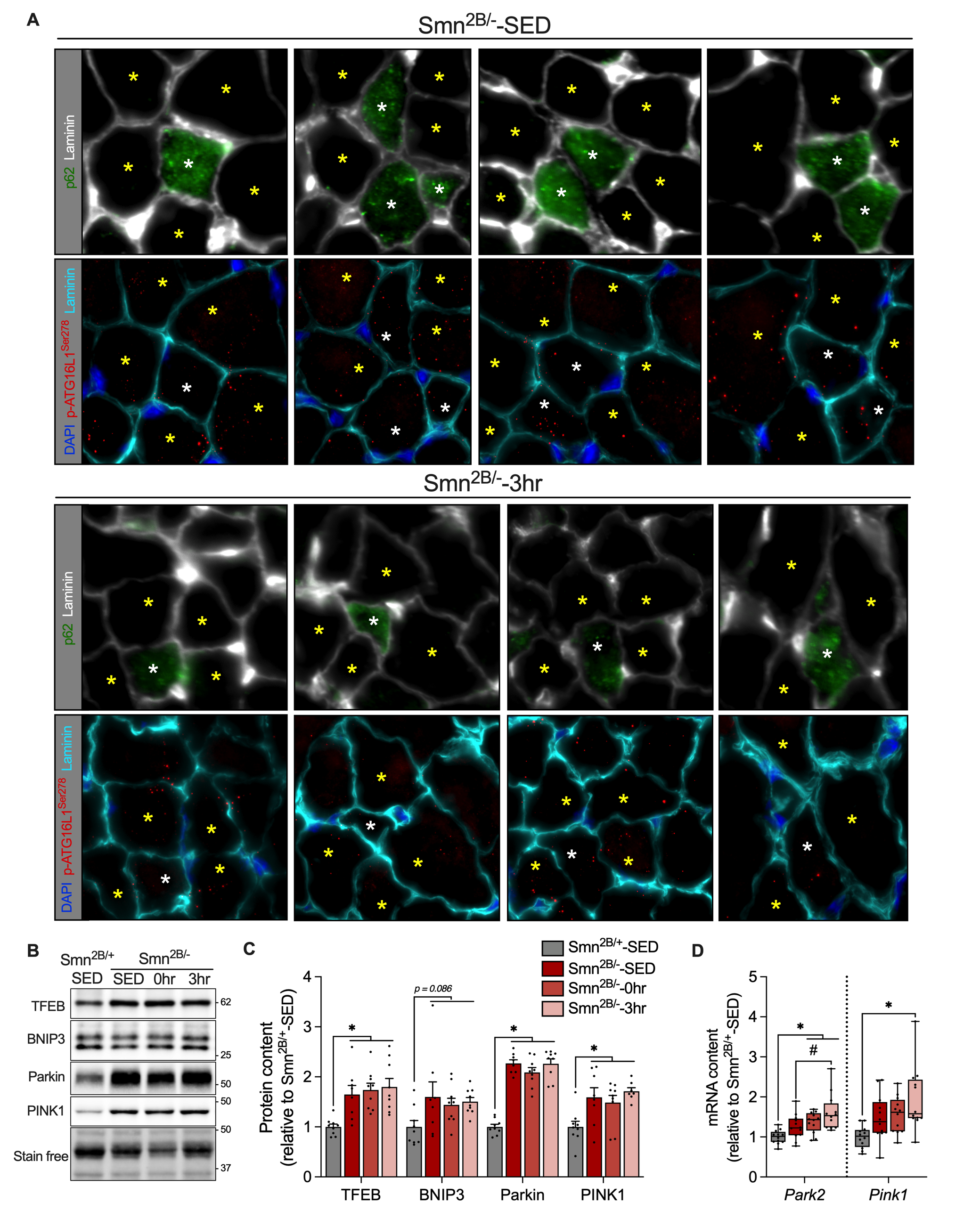


**Figure S3. Acute exercise does not alter the abundance of mitophagy-related protein in skeletal muscle of SMA mice. (A)** Immunofluorescence microscopy of (top panel) p62 (green) and laminin (white), as well as (bottom panel) p-ATG16L1^Ser278^ puncta (red), DAPI (blue) and laminin (cyan) in EDL serial cross-sections of mice from *Smn^2B/-^*-SED, and *Smn^2B/-^*-3hr animals. White asterisk denotes p62 positive fibers and yellow asterisk identify neighbouring p62 negative fibers. **(B)** Western blots of TFEB, BNIP3, Parkin, and PINK1 protein expression. A stain-free blot displayed below shows sample loading. Approximate molecular weights (kDa) shown at right of blots. **(C)** Graphical summaries of TFEB, BNIP3, Parkin and PINK1 protein content. n = 8-9. **(D)** mRNA levels of *Park2*, and *Pink1* in TA muscles of mice in the four experimental groups. n = 11-12. Data are expressed relative to the *Smn^2B/+^*-SED group and are means ± SEM with individual data points displayed. * p < 0.05 vs. WT-SED, and # p < 0.05 vs. *Smn^2B/-^*-SED, one-way ANOVA.

**
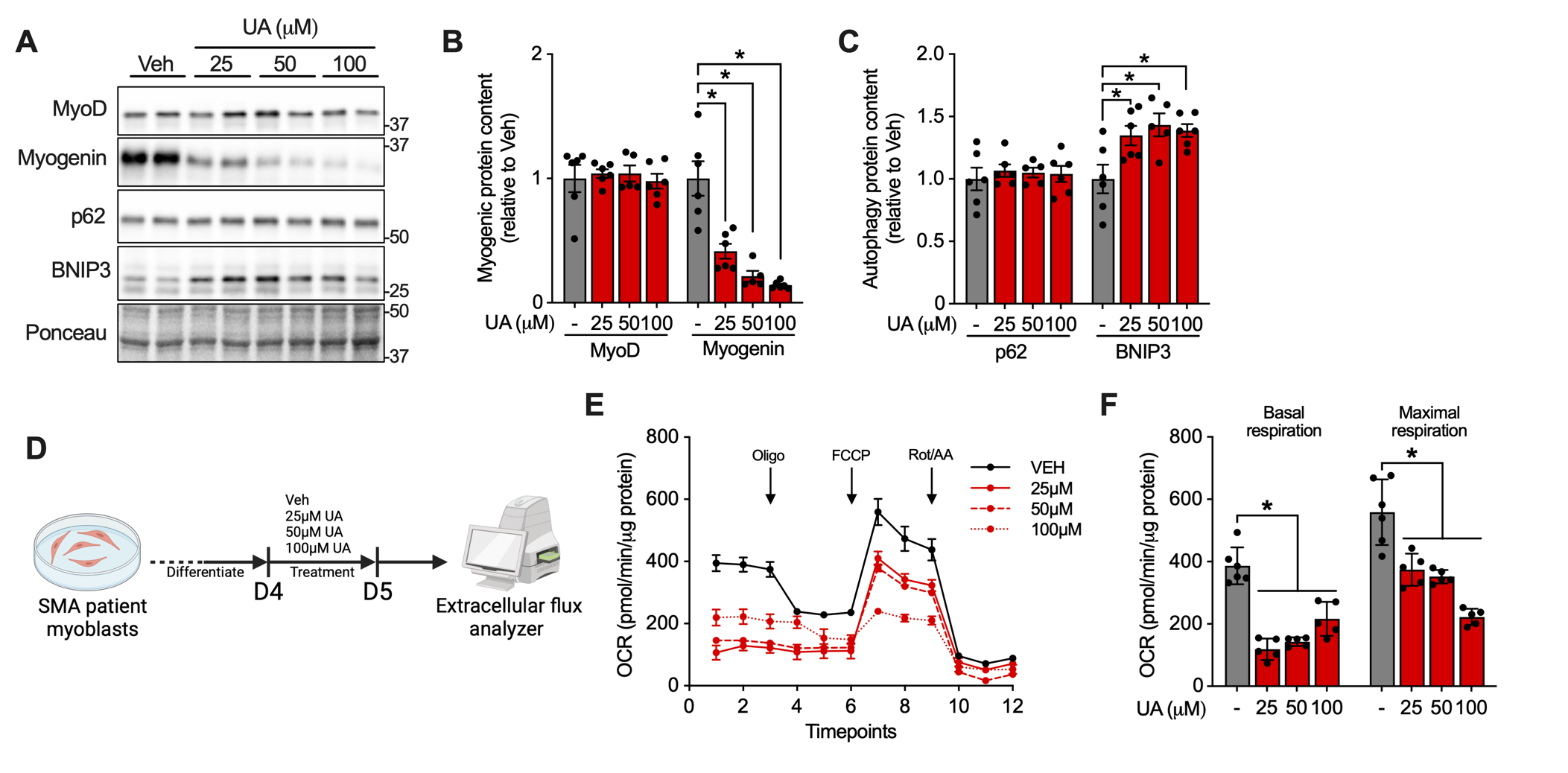
**

**Figure S4. Myogenic protein expression and cellular respiration of SMA myotubes immediately after Veh or UA treatment (A)** Typical Western blots of myoblast differentiation protein 1 (MyoD), myogenin, p62 and BNIP3 proteins in SMA patient-derived myotubes following 24hrs of Veh or UA administration. A Ponceau S stain displayed below shows sample loading. Approximate molecular weights (kDa) shown at right of blots. Graphical summaries of **(B)** myogenic proteins including MyoD, and myogenin as well as **(C)** autophagy-related proteins such as p62 and BNIP3. **(D)** Visual schematic of the experimental design to measure OCR in SMA patient-derived myotubes immediately after 24 hrs of Veh or UA treatment. **(E)** Cellular respiration tracings in response to the addition of Oligo, FCCP, Rot and AA. **(F)** Summary of basal and maximal OCR in SMA myotubes normalized to total protein content. n = 5-6 technical replicates for each treatment condition. Data are expressed relative to Veh and are means ± SEM with individual data points displayed. * p < 0.05 vs. Veh, one-way ANOVA.

**SUPPLEMENTARY REFERENCES**

S1. Carlini MJ, Triplett MK, Pellizzoni L. (2022) Neuromuscular denervation and deafferentation but not motor neuron death are disease features in the Smn2B/- mouse model of SMA. PLoS One 17, e0267990

S2. Sirca, A. and Kostevc, V. (1985) The fibre type composition of thoracic and lumbar paravertebral muscles in man. J Anat 141, 131

S3. Savage, K.J. and McPherron, A.C. (2010) Endurance exercise training in myostatin null mice. Muscle Nerve 42, 355

S4. Call JA, McKeehen JN, Novotny SA, Lowe DA. (2010) Progressive resistance voluntary wheel running in the mdx mouse. Muscle Nerve 42, 871

S5. Bittel AJ, Bittel DC, Gordish-Dressman H, Chen YW. (2024) Voluntary wheel running improves molecular and functional deficits in a murine model of facioscapulohumeral muscular dystrophy. iScience 27, 108632

S6. Soendenbroe C, Karlsen A, Svensson RB, Kjaer M, Andersen JL, Mackey AL. (2024) Marked irregular myofiber shape is a hallmark of human skeletal muscle ageing and is reversed by heavy resistance training. J Cachexia Sarcopenia Muscle 15, 306–318

S7. Murray LM, Beauvais A, Gibeault S, Courtney NL, Kothary R. (2015) Transcriptional profiling of differentially vulnerable motor neurons at pre-symptomatic stage in the Smn (2b/-) mouse model of spinal muscular atrophy. Acta Neuropathol Commun. 3, 55

S8. Norlin S, Axelsson J, Ericsson M, Edlund H. (2023) O304 ameliorates hyperglycemia in mice by dually promoting muscle glucose effectiveness and preserving β-cell function. Commun Biol 6, 1–16

S9. Andrzejewski S, Gravel SP, Pollak M, St-Pierre J. (2014) Metformin directly acts on mitochondria to alter cellular bioenergetics. Cancer Metab 2, 1–14

S10. Deguise MO, Pileggi C, De Repentigny Y, Beauvais A, Tierney A, Chehade L *et al*. (2021) SMN Depleted Mice Offer a Robust and Rapid Onset Model of Nonalcoholic Fatty Liver Disease. Cell Mol Gastroenterol Hepatol 12, 354–377

S11. Triolo M, Bhattacharya D, Hood DA. (2022) Denervation induces mitochondrial decline and exacerbates lysosome dysfunction in middle-aged mice. Aging 14, 8900–8913

S12. Webb EK, Ng SY, Mikhail AI, Stouth DW, vanLieshout TL, Syroid AL *et al*. Impact of short-term, pharmacological CARM1 inhibition on skeletal muscle mass, function, and atrophy in mice. Am J Physiol Endocrinol Metab 325, E252-E266

S13. Stouth DW, vanLieshout TL, Ng SY, Webb EK, Manta A, Moll Z *et al*. (2020) CARM1 Regulates AMPK Signaling in Skeletal Muscle. iScience 23

S14. Bernabò P, Tebaldi T, Groen EJN, Lane FM, Perenthaler E, Mattedi F *et al*. (2017) In Vivo Translatome Profiling in Spinal Muscular Atrophy Reveals a Role for SMN Protein in Ribosome Biology. Cell Rep 21, 953–965

S15. Valsecchi V, Errico F, Bassareo V, Marino C, Nuzzo T, Brancaccio P *et al*. (2023) SMN deficiency perturbs monoamine neurotransmitter metabolism in spinal muscular atrophy. Commun Biol 6, 1155

S16. Moore TM, Zhou Z, Cohn W, Norheim F, Lin AJ, Kalajian N *et al*. (2019) The impact of exercise on mitochondrial dynamics and the role of Drp1 in exercise performance and training adaptations in skeletal muscle. Mol Metab 21, 51

**SUPPLEMENTARY METHODS**

*Exercise protocol and tissue collection. Smn^2B/+^* and *Smn^2B/-^* mice were randomly assigned to either a SED or exercise group. Animals in the exercise groups were acclimatized to a motorized treadmill (Columbus Instruments, Columbus, OH, USA) at P15 and P16 for 5 min at a speed of 3 m/min. On P17, animals were administered a single dose of exercise at a constant speed of 3 m/min at a 0° incline until the inability to continue running was empirically determined. The exercise endpoint was defined when mice were no longer responsive to gentle mechanical prodding with a brush, and were unable to self-right from a supine position within 30 sec. Following the cessation of physical activity, animals were euthanized, and tissues were harvested immediately after (*Smn^2B/-^*-0hr) or following a 3hr recovery period (*Smn^2B/-^*-3hr). The acute exercise response in healthy skeletal muscle has been extensively studied [1–3] and therefore, we omitted *Smn^2B/+^* mice from most analyses and focused on the novelty of further investigating the effects of a single dose of exercise in SMA-like mice as we have done previously [4]. All samples were collected at the same time of day, between 10:00 and 14:00.

*Cell culture and urolithin A treatment.* SMA cells were plated on a 6-well plate at a density of 10,000 cells/cm^2^ and grown in the presence of skeletal muscle GM (C23160, PromoCell, Heidelberg, Germany). The cells were maintained in a humidified incubator at 5% CO_2_ and 37°C. Myogenic differentiation was induced at ∼100% cell confluency, after which GM was replaced with DM consisting of DMEM/F12 (11330057; Thermo Fisher Scientific, Waltham, MA, USA) supplemented with 2% horse serum (16060122, Thermo Fisher Scientific). For UA experiments, D4 differentiated myotubes were treated with DMSO or UA (25, 50 or 100 μM) dissolved in DMSO (0.08% final concentration) for 24 hrs and processed for protein and immunofluorescence analysis. For autophagy flux experiments, D4 SMA myotubes were treated with Veh or 50 μM of UA for a total of 24 hrs. During the latter 12 hrs, cells were co-incubated with either 100 μM of CQ to inhibit autophagy or Veh (ddH_2_O). Following the 24-hr treatment, cells were immediately flash frozen, and protein was isolated for Western blotting. LC3 II and p62 flux was determined based on the difference in protein densitometry between CQ and Veh conditions for each respective treatment (i.e., DMSO Veh or 50 μM UA). All experiments were performed with an n = 6 technical replicates for each treatment condition.

*Protein extraction and Western blotting.* TRI muscles were placed in RIPA buffer (1:20, Sigma-Aldrich, St. Louis, MO, USA) supplemented with protease and phosphatase inhibitors (Roche, Mississauga, Ontario, Canada) and homogenized using steel ball bearings and a motorized tissue lyser (Qiagen, Hilden, Germany). For protein extraction from myotubes, cells were sonicated in 100 μL of RIPA buffer supplemented with inhibitors. The supernatant was collected after homogenates and cell lysates were centrifuged at 14,000 g for 15 min, and protein concentrations were determined using a standard bicinchoninic protein assay (BCA; Thermo Fisher Scientific). For Western blotting experiments, proteins (~20 μg) were separated on a 4-20% Criterion TGX precast gel (Bio-Rad Laboratories, Mississauga, ON, Canada) and transferred onto nitrocellulose membranes (Bio-Rad Laboratories) using a Trans-Blot Turbo transfer system (Bio-Rad Laboratories). Stain-free membranes were then imaged and placed in blocking solution (5% BSA in 1X TBST) for 1 hr prior to an overnight incubation at 4 °C with primary antibodies listed in Table S1. Blots were then washed in 1X TBST for 3 x 5 min and incubated in the appropriate secondary antibody (Table S1) for 1 hr at room temperature. Next, membranes were washed in 1X TBST buffer for 3 x 5 min prior to applying luminol based enhanced chemiluminescence reagent (Bio-Rad Laboratories) for visualization. Finally, proteins were imaged using the ChemiDoc Imaging System (Bio-Rad Laboratories) and quantified using Image Lab software (Bio-Rad Laboratories). All bands were normalized to their respective stain-free intensity prior to analysis.

*RNA isolation and purification.* TA muscle samples were homogenized using 1 mL of TRIzol reagent (Invitrogen, Carlsbad, CA, USA) in Lysing D Matrix tubes (MP Biomedicals, Solon, OH, USA). Samples were shaken vigorously for 15 sec with chloroform, incubated for 5 min at room temperature and centrifuged at 12,000 g for 10 min. The RNA phase was purified using the total RNA Omega Bio-Tek kit (VWR International, Radnor, PA, USA) and RNA concentration was determined using a NanoDrop 1000 Spectrophotometer (Thermo Fisher Scientific). All samples were adjusted to a concentration of 200 ng/µL and reverse transcribed using a high-capacity cDNA reverse transcription kit (Thermo Fisher Scientific) as instructed by the manufacturer.

*Quantitative real-time polymerase chain reaction (qPCR).* All samples were run in triplicate on a 384-well plate with each reaction containing 2 μg of cDNA and GoTaq qPCR Master Mix (Promega, Madison, WI, USA). Gene expression was determined using the comparative *C_T_* method [5]. Mean mRNA expression of *glyceraldehyde 3-phosphate dehydrogenase* (*Gapdh*) and *ribosomal protein S11* (*Rps11*) did not differ between genotypes or in response to exercise (data not shown) and were therefore used as housekeeping genes. All qPCR primers used are listed in Table S2.

*End-point PCR.* For detection of *Opa1 +/-* exon (ex) 4b alternative splicing, cDNA was added to a reaction containing Taq polymerase and primers (listed in Table 2). PCR products were resolved on a 2% agarose gel at 120 mV for 1 hr. Percent inclusion of ex 4b was determined as the intensity of the *Opa1* +ex4b band relative to the total intensity of *Opa1* +ex4b plus *Opa1* -ex4b bands. Analysis was done using Image Lab software (Bio-Rad Laboratories).

*Histochemical staining.* SDH staining was performed on 10 μm thick EDL cross-sections as previously outlined [6]. In brief, muscle sections were immersed in SDH incubation buffer (0.2 M sodium succinate, 0.2 M phosphate buffer, pH 7.4, and nitro blue tetrazolium) at 37 °C for 1 hr then rinsed with distilled water prior to being sequentially dehydrated in 30%, 60%, 90% acetone. Finally, slides were mounted using Permount and imaged at 20x magnification using Nikon 90i eclipse upright microscope (Nikon Instruments). Mean SDH intensity was determined using Image J software by manually circling an average of ~260 ± 16 fibers per sample. For low and high SDH-specific myofiber size analysis, fibers within each sample were ranked based on mean SDH intensity and the average minimum ferret diameter was calculated for the highest and lowest 30 myofibers from P21 *Smn^2B/+^* and *Smn^2B/-^*.

*Immunofluorescence microscopy and analysis.* Visualization of newly forming autophagosomes via p-ATG16L1^Ser278^ was carried out as previously described [7,8]. Briefly, cryo-sections were fixed in 4% PFA, followed by a 10 min incubation in 3% hydrogen peroxide and then blocked in 5% BSA in TBST. Slides were then incubated overnight with primary antibodies p-ATG16L1^Ser278^ and laminin (Table S1). Samples were then washed in 1X PBS buffer before the appropriate secondary antibody was applied (Table S1) for 2 hrs at room temperature. Slides were once again washed in 1X PBS and then 4′,6-diamidino-2-phenylindole dihydrochloride (DAPI; 1:20,000; D9542; Sigma-Aldrich) was applied for 5 min to label nuclei. Finally, slides were washed in 1X PBS, mounted and imaged on a 60x oil objective using the Nikon Elements Microscopic Imaging software (Nikon Instruments Inc, Melville, NY, USA). For each sample, ~6 ± 2 field of views (FOVs) were captured and the NIS thresholding feature was utilized to detect individual p-ATG16L1^Ser278^ puncta. The number of objects (i.e., puncta) per FOV was acquired and normalized to the total FOV area to calculate number of puncta per 1,000 μm^2^. Laminin staining was also used to assess myofiber size by manually outlining the borders of 300 muscle fibers per sample using Nikon Elements software. Shape factor index was used as a proxy for an irregular myofiber architecture and was computed as previously described using myofiber cross-sectional area and perimeter [9].

Staining for cytoplasmic p62 was carried out as previously done [10]. EDL cross-sections were fixed in 4% PFA for 6 min, neutralized in 0.1 M glycine (pH 7.4) for 2 x 15 min, blocked for 1.5 hrs in blocking solution (3% BSA, 40 ug/mL Fab anti-bouse IgG and 0.25% Triton-X) and then incubated overnight at 4 °C in p62 and laminin antibodies (Table S1). Finally, samples were incubated in the appropriate secondary antibodies and washed in PBS prior to mounting with Prolong Gold antifade reagent. Images of the muscle cross-section were captured using a 60x oil objective and the Nikon Elements Microscopic Imaging software (Nikon Instruments Inc). All fibers expressing cytoplasmic p62 were counted and made relative to the total number of myofibers.

For myotube staining, differentiated cells were rinsed in 1X PBS and incubated in 4% PFA for 10 min. After fixation, myotubes were washed in 1X PBS for 3 x 5 min prior to permeabilization in 0.1% Triton-X for 20 min. A blocking solution (2% BSA in 1X PBS) was then applied for 1 hr at room temperature followed by an overnight incubation in myosin heavy chain I (MHC I; neat, Table S1) at 4 °C. Next, cells were once again rinsed in 1X PBS for 3 x 5 min and the appropriate secondary antibody was applied for 2 hrs at room temperature. A total of 5 FOVs per well were captured on a 10x objective using the Nikon Elements Microscopic Imaging software (Nikon Instruments Inc, Melville, NY, USA).

*Transmission electron microscopy (TEM) and analysis.* TA samples from *Smn^2B/+^* and *Smn^2B/-^* mice were processed for TEM as previously described [11]. Note that the data relating to mitochondrial morphology was generated from images that were previously acquired by Deguise et al [11]. Quantitative measurements of mitochondrial morphology were performed using Image J software as previously done by others [12]. Roughly 8 ± 2 FOVs at 15,000x magnification were analyzed for 3 biologically unique samples per group. Aspect ratio was computed as [(maximum Feret’s diameter)/(minimum Feret’s diameter)] and reflects mitochondrial elongation. Form factor [(perimeter^2^)/(4π·surface area)] represents the complexity and branching of the organelle reticulum. Circularity [4π·(surface area/perimeter^2^)] and roundness [4·(surface area)/(π·major axis^2^)] are two-dimensional indicators of sphericity and fragmentation with a value of 1 indicating a perfect circle.

Metabolic measurements of human immortalized SMA myotubes. Cells were seeded at a density of 60,000 per well with 250 μL of myoblast GM and incubated overnight. The following day, cells were washed in PBS and myogenic differentiation was initiated by switching to DM. SMA myotubes were allowed to differentiate for 3 or 4 days prior to a 24-hr treatment with DMSO, or UA (25 μM, 50 μM, 100 μM). OCR measurements were performed on day 5 of differentiation for all experimental procedures. Briefly, muscle cells were switched to Seahorse assay media consisting of sterile H_2_O, powdered DMEM (Sigma-Aldrich, D5030), sodium pyruvate (1 mM, Sigma-Aldrich, S8636), L-glutamine (2 mM, Gibco, 25030081), sodium chloride (1.85 g/L, BioShop, SOD004.1) and glucose (25 mM, Sigma-Aldrich, G7021). Cellular respiration was evaluated using 1.5 μM of oligomycin A (ATP-synthase inhibitor; Sigma-Aldrich, 75351), 2 μM of carbonyl cyanide-p-trifluoromethoxyphenylhydrazone (FCCP; an uncoupler of mitochondrial oxidative phosphorylation; Sigma-Aldrich, C2920) as well as 0.5 μM each of rotenone (Complex I inhibitor; Sigma-Aldrich, R8875) and antimycin A (Complex III inhibitor; Sigma-Aldrich, A8674). Following the completion of the assay, all values were normalized to total protein concentration determined through a BCA assay (ThermoFischer Scientific).

| **Table S1:** List of primary antibodies | | | | | |
| --- | --- | --- | --- | --- | --- |
| **Western Blotting** | | | | | |
| **Protein** | **Manufacture** | **Product #** | **Host** | **Primary Dilution** | **Secondary Dilution** |
| p-AMPK^Thr 172^ | CST | 2535 | Rabbit | 1:1000 | 1:10,000 |
| t-AMPK | CST | 2532 | Rabbit | 1:1000 | 1:10,000 |
| BNIP3 | CST | 3769 | Rabbit | 1:1000 | 1:10,000 |
| Citrate synthase | Abcam | ab96600 | Rabbit | 1:1000 | 1:10,000 |
| p-DRP1^S616^ | CST | 3455 | Rabbit | 1:1000 | 1:10,000 |
| p-DRP1^S637^ | CST | 4867 | Rabbit | 1:1000 | 1:10,000 |
| t-DRP1 | CST | 8570 | Rabbit | 1:1000 | 1:10,000 |
| FIS1 | Proteintech | 10956-1-AP | Rabbit | 1:1000 | 1:10,000 |
| LC3 I/II | CST | 4108 | Rabbit | 1:1000 | 1:10,000 |
| MFN-2 | Abcam | ab56889 | Mouse | 1:1000 | 1:10,000 |
| OPA1 | Abcam | ab42364 | Rabbit | 1:1000 | 1:10,000 |
| OXPHOS | Abcam | ab110413 | Mouse | 1:1000 | 1:10,000 |
| Parkin | CST | 2132 | Rabbit | 1:1000 | 1:10,000 |
| PGC-1α | EMD Millipore | AB3242 | Rabbit | 1:1000 | 1:10,000 |
| PINK1 | Novus Biologicals | BC100-494 | Rabbit | 1:1000 | 1:10,000 |
| p62 | Sigma-Aldrich | P0067 | Rabbit | 1:1000 | 1:10,000 |
| TFEB | Bethyl Labs. | A303-673A | Rabbit | 1:1000 | 1:10,000 |
| p-ULK1^Ser555^ | CST | 5869 | Rabbit | 1:1000 | 1:10,000 |
| t-ULK1 | CST | 8054 | Rabbit | 1:1000 | 1:10,000 |
| **Immunofluorescence** | | | | | |
| **Protein** | **Manufacture** | **Product #** | **Host** | **Primary Dilution** | **Secondary Dilution** |
| p-ATG16L1^Ser278^ | Abcam | ab195242 | Rabbit | 1:300 | 1:500 |
| Laminin | Sigma-Aldrich | L0663 | Rat | 1:500 | 1:500 |
| MHC I | DSHB | A4.951 | Mouse | Neat | 1:500 |
| p62 | Progen Biotechnik | GP62-C | Guinea pig | 1:300 | 1:500 |
| CST, Cell Signaling Technologies; DSHB, Developmental Studies Hybridoma Bank. | | | | | |

| **Table S2:** PCR primer sequences | |
| --- | --- |
| **Gene** | **Primer sequence** |
| *Bnip3 – F* | TTCCACTAGCACCTTCTGATGA |
| *Bnip3 – R* | GAACACCGCATTTACAGAACAA |
| *Chrng – F* | ACGAAGGCCTGTGGATATTG |
| *Chrng – R* | ACAGAGATGGAGCAGGAGGA |
| *Cox – F* | CTCCAACGAATGGAAGACAG |
| *Cox – R* | TGACAACCTTCTTAGGGAAC |
| *Cs – F* | TTGTTTTGTTTCAGGGGCCTT |
| *Cs - R* | GTGAACTGGTGAGGGGAGAAG |
| *Dnml1 –* F | CCTCAGATCGTCGTAGTGGGA |
| *Dnml1 –* R | GTTCCTCTGGGAAGAAGGTCC |
| *Fis1 –* F | TGTCCAAGAGCACGCAATTTG |
| *Fis1 –* R | CCTCGCACATACTTTAGAGCCTT |
| *Gapdh –* F | ﻿AACACTGAGCATCTCCCTCA |
| *Gapdh* – R | ﻿GTGGGTGCAGCG AACTTTAT |
| *Map1lc3 – F* | CACTGCTCTGTCTTGTGTAGGTTG |
| *Map1lc3 – R* | TCGTTGTGCCTTTATTAGTGCATC |
| *Mfn2 –* F | GCTCAGGAGCAGCGGGTTTA |
| *Mfn2 –* R | TGTGGACACCTGCCTTTCCA |
| *Myog – F* | TCACATAAGGCTAACACCCAG |
| *Myog – R* | GGAATTCGAGGCATATTATGA |
| *Ncam – F* | GGTGACCCCTGATTCAGAAA |
| *Ncam – R* | GGATGGAGAAGACGGTGTGT |
| *Nrf2 – F* | TTCTTTCAGCAGCATCCTCTCCAC |
| *Nrf2 – R* | ACAGCCTTCAATAGTCCCGTCCAG |
| *Opa1 –* F | CAGAGGATGGTGCTCGTGGA |
| *Opa1 –* R | TCCGTCTTGGATGCACAGGA |
| *Opa1 exon 4b –* F | TTGGCCAGCAAGGTTAGCTGCAAG |
| *Opa1 exon 4b –* R | TGCTTGTCACTTTCAGATCCATGA |
| *Park2 –* F | TCTTCCAGTGTAACCACCGTC |
| *Park2 –* R | GGCAGGGAGTAGCCAAGTT |
| *Pink1 –* F | GTGGAACATCTCGGCAGGTT |
| *Pink1 –* R | CCTCTCTTGGATTTTCTGTAAGTGAC |
| *Rps11 – F* | ﻿CGTGACGAACATGAAGATGC |
| *Rps11 – R* | ﻿GCACATTGAATCGCACAGTC |
| *Sirt1 – F* | GGAACCTTTGCCTCATCTACA |
| *Runx1 – F* | CCGCAGCATGGTGGAGGTA |
| *Runx1 – R* | AGCGATGGGCAGGGTCTTG |
| *Sirt1 – R* | CACCTAGCCTATGACACAACTC |
| *Sqstm1 – F* | CCCAGTGTCTTGGCATTCTT |
| *Sqstm1 – R* | AGGGAAAGCAGAGGAAGCTC |
| *Tfam – F* | TAGGCACCGTATTGCGTGAG |
| *Tfam – R* | GTGCTTTTAGCACGCTCCAC |
| *Tp53 – F* | CCGACCTATCCTTACCATCATC |
| *Tp53 – R* | TTCTTCTGTACGGCGGTCTC |

**REFERENCES**

1. Hood, DA, Memme JM, Oliveira AN, Triolo M. (2019) Maintenance of Skeletal Muscle Mitochondria in Health, Exercise, and Aging. *Annu Rev Physiol.* 81, 19–41

2. Hood DA, Uguccioni G, Vainshtein A, D’souza D*.* (2011) Mechanisms of exercise-induced mitochondrial biogenesis in skeletal muscle: implications for health and disease. *Compr Physiol* 1, 1119–1134

3. Drake JC, Wilson RJ, Yan Z*.* (2016) Molecular mechanisms for mitochondrial adaptation to exercise training in skeletal muscle. *FASEB J*, 30, 13–22

4. Ng SY, Mikhail A, Ljubicic V*.* (2019) Mechanisms of exercise-induced survival motor neuron expression in the skeletal muscle of spinal muscular atrophy-like mice. *J Physiol.* 587, 4757–4778

5. Schmittgen, T.D. and Livak, K.J. (2008) Analyzing real-time PCR data by the comparative CT method. *Nat Protoc* 3, 1101

6. Mikhail AI, Nagy PL, Manta K, Rouse N, Manta A, Ng SY *et al.* (2022) Aerobic exercise elicits clinical adaptations in myotonic dystrophy type 1 patients independent of pathophysiological changes. *J Clin Invest.* 132, e156125

7. Tian W, Alsaadi R, Guo Z, Kalinina A, Carrier M, Tremblay ME *et al.* (2020) An antibody for analysis of autophagy induction. *Nat Methods* 17, 232–239

8. Mikhail AI, Manta A, Ng SY, Osborne AK, Mattina SR, Mackie MR *et al.* (2023) A single dose of exercise stimulates skeletal muscle mitochondrial plasticity in myotonic dystrophy type 1. *Acta Physiol (Oxf).* 237, e13943

9. Soendenbroe C, Karlsen A, Svensson RB, Kjaer M, Andersen JL, Mackey AL. (2024) Marked irregular myofiber shape is a hallmark of human skeletal muscle ageing and is reversed by heavy resistance training. *J Cachexia Sarcopenia Muscle* 15, 306–318

10. Ham DJ, Börsch A, Chojnowska K, Lin S, Leuchtmann AB, Ham AS *et al.* (2022) Distinct and additive effects of calorie restriction and rapamycin in aging skeletal muscle. *Nat Commun* 13

11. Deguise MO, Boyer JG, McFall ER, Yazdani A, De Repentigny Y, Kothary R*.* (2016) Differential induction of muscle atrophy pathways in two mouse models of spinal muscular atrophy. *Sci Rep* 6, 28846

12. Picard M, Gentil BJ, McManus MJ, White K, St Louis K, Gartside SE *et al.* (2013) Acute exercise remodels mitochondrial membrane interactions in mouse skeletal muscle. *J Appl Physiol* 115, 1562–1571
